# Discovery of a Flexible Triazolylbutanoic Acid as a Highly Potent Uric Acid Transporter 1 (URAT1) Inhibitor

**DOI:** 10.3390/molecules21111543

**Published:** 2016-11-16

**Authors:** He Tian, Wei Liu, Zhixing Zhou, Qian Shang, Yuqiang Liu, Yafei Xie, Changying Liu, Weiren Xu, Lida Tang, Jianwu Wang, Guilong Zhao

**Affiliations:** 1 School of Chemistry and Chemical Engineering, Shandong University, Jinan 250100, China; tian_he9562@hotmail.com; 2 Tianjin Key Laboratory of Molecular Design and Drug Discovery, Tianjin Institute of Pharmaceutical Research, Tianjin 300193, China; liuw@tjipr.com (W.L.); zhouzx@tjipr.com (Z.Z.); shangq@tjipr.com (Q.S.); liuyq@tjipr.com (Y.L.); xieyf@tjipr.com (Y.X.); liucy@tjipr.com (C.L); xuwr@tjipr.com (W.X.); tangld@tjipr.com (L.T.)

**Keywords:** gout, hyperuricemia, URAT1 inhibitor, lesinurad, synthesis

## Abstract

In order to systematically explore and understand the structure–activity relationship (SAR) of a lesinurad-based hit (**1c**) derived from the replacement of the S atom in lesinurad with CH_2_, 18 compounds (**1a**–**1r**) were designed, synthesized and subjected to in vitro URAT1 inhibitory assay. The SAR exploration led to the discovery of a highly potent flexible URAT1 inhibitor, **1q**, which was 31-fold more potent than parent lesinurad (IC_50_ = 0.23 μM against human URAT1 for **1q** vs 7.18 μM for lesinurad). The present study discovered a flexible molecular scaffold, as represented by **1q**, which might serve as a promising prototype scaffold for further development of potent URAT1 inhibitors, and also demonstrated that the S atom in lesinurad was not indispensable for its URAT1 inhibitory activity.

## 1. Introduction

Gout, which is characterized by recurrent joint swelling and pain, is the most prevalent form of inflammatory arthritis. It is caused by the deposition of monosodium urate (MSU) in joints and soft tissues [1]. If left untreated or inadequately managed, gout will lead to permanent joint destruction, bone erosion, and kidney impairment, dramatically affecting patients’ quality of life and even threatening their lives [2]. More recently, a deeper understanding of the gout pathophysiology has led to the appreciation that gout impacts patients with consequences well beyond the episodes of acute inflammatory arthritis, and multiple lines of evidence have been accumulated to demonstrate that hyperuricemia was the independent risk factor for hypertension, hyperlipidemia, diabetes, cardiovascular diseases, etc. [3,4]. Persisted hyperuricemia is the prerequisite of MSU formation and deposition, which is defined as the elevation of serum uric acid (sUA) levels above the saturation point of MSU in the fluid at physiological pH and temperature, i.e., 6.8 mg/dL (404 μmol/L) [1]. Approximately 98% of the uric acid (pH = 5.75) is in the ionized form, i.e., urate anion, in the extracellular compartment (pH = 7.4), and due to the existence of a high concentration of sodium ion in the extracellular compartment, urate largely presents as MSU [2,5]. Gout is one of the earliest diseases recognized by physicians in history [6]. The last few decades have witnessed the rising prevalence of gout and hyperuricemia in both developed and developing countries [7,8,9], which presents significant burdens on the individual and community in many respects [10,11].

Acute gout attacks have been traditionally treated with colchicine, nonsteroidal anti-inflammatory drugs (NSAIDs), and corticosteroids, agents that often reduce gout flares but that are also associated with many adverse effects [12]. However, the fundamental of treatment of gout is to dissolve MSU crystals by urate-lowering therapy (ULT) with xanthine oxidase inhibitors (XOIs) such as allopurinol and febuxostat, uricosuric agents such as probenecid, sulfinpyrazone, and benzbromarone, and uricase such as pegloticase [13,14]. The target of ULT is to maintain sUA < 6 mg/dL (360 μmol/L) and even <5 mg/dL (300 μmol/L) for severe gout, as the guidelines recommend [1,2]. XOIs are usually used as first-line ULT, albeit with numerous severe adverse effects and often with low response rates, and when patients are refractory to or contraindicated for XOIs, uricosuric agents are used as second-line therapy [2,15]. Uricases are only indicated for patients with severe gout, such as those with tophaceous deformities and complications, which, however, are associated with infusion reactions and loss of urate-lowering efficacy within a few months of treatment due to the development of antibodies [15].

**Figure 1:**
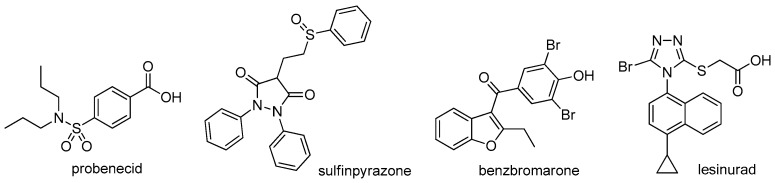
Structures of approved URAT1 inhibitors.

**Figure 2:**
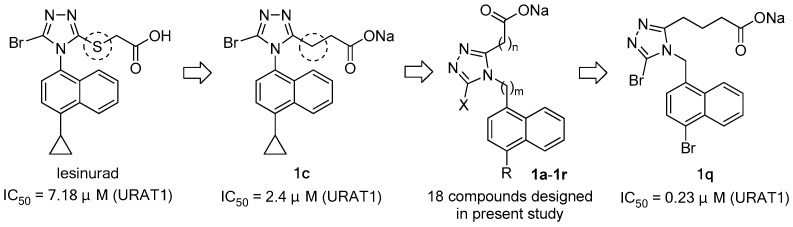
Design of URAT1 inhibitors in present study and discovery of **1q**.

Unlike in other species, in humans and in most non-human primates, uric acid is the final metabolite of dietary and endogenous purine metabolism. Humans lack uricase, an enzyme capable of converting uric acid to allantoin, which is 5–10 times more water-soluble than uric acid and is therefore much more readily eliminated [2,16]. Hyperuricemia is caused by over-production and/or under-excretion of urate, and among the patients with hyperuricemia, 90% of them are urate under-excretors, while 10% are over-producers [1]. Approximately one-third of urate is excreted via the gastrointestinal tract, and the remaining two-thirds via kidney [2]. Most of the urate filtered in kidney is reabsorbed, and this process is mainly mediated by uric acid transporter 1 (URAT1), also known as urate-anion exchanger 1, which is prominently expressed in epithelial cells of proximal tubules in the renal cortex [17]. Given the fact that 90% of the patients with hyperuricemia are urate under-excretors, URAT1 inhibitors, which lower sUA by inducing uricosuria, were believed to be a very promising class of uricosuric agents for the treatment of hyperuricemia and gout [18]. A number of uricosuric agents that had already been approved were later found to be UART1 inhibitors after the identification of URAT1 in 2002 (Figure 1) [17]; however, these agents are associated with a variety of severe adverse effects or drawbacks, such as severe hepatotoxicity of benzbromarone, drug interactions of probenecid with NSAIDs, penicillin, heparin, etc., and limited efficacy of probenecid and sulfinpyrazone [15,17]. Besides, some of them are not available in specific countries. For example, benzbromarone was withdrawn in Europe in 2003 and has never been approved in the US because of severe hepatotoxicity. Lesinurad, a novel URAT1 inhibitor developed by Ardea Biosciences and AstraZeneca, was approved by the FDA at the end of 2015 for the treatment of hyperuricemia associated with gout in combination with an XOI [19]. In a study on lesinurad derivatives as URAT1 inhibitors in our laboratories, we were surprised to find that compound **1c**, the sodium salt of **10b** derived from the replacement of the S atom in lesinurad with CH_2_, was three-fold more potent than parent lesinurad in in vitro URAT1 inhibitory assay (Figure 2). This preliminary finding suggested that the S atom might not be indispensable and its replacement with CH_2_ could even increase the URAT1 inhibitory activity. Encouraged by this preliminary finding and promising hypothesis, we carried out a systematic structure–activity relationship (SAR) exploration of this interesting molecular scaffold, with the expectation of a better understanding of its SAR. Fortunately, the systematic SAR exploration led to the discovery of **1q** as a highly potent URAT1 inhibitor, which was 31-fold more active than parent lesinurad in in vitro URAT1 inhibitory assay (IC_50_ = 0.23 μM against human URAT1 for **1q** vs. 7.18 μM for lesinurad).

## 2. Results and Discussion

### 2.1. Chemistry

**Scheme 1:**
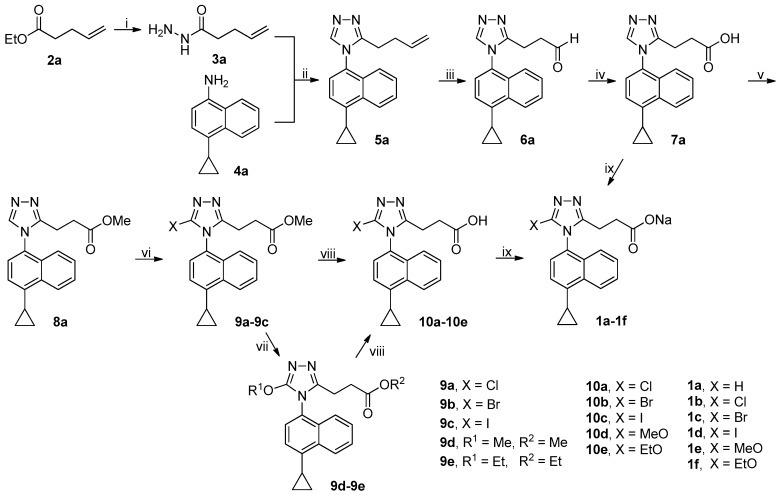
Synthetic route to **1a**–**1f**. *Reagents and conditions*: (i) 80% N_2_H_4_·H_2_O, MeOH, r.t.; (ii) (a) DMFDMA, **3a**, MeCN, 50 °C, open vessel; (b) **4a**, AcOH, reflux; (iii) (a) NMMO, OsO_4_, THF/H_2_O (4/1), r.t.; (b) NaIO_4_, THF/H_2_O (4/1), r.t.; (iv) NaClO_2_, NaH_2_PO_4_, 2-methyl-2-butene, *t*-BuOH/H_2_O (4/1), 0 °C-rt; (v) MeOH, EDCI, DMAP, CH_2_Cl_2_, 0 °C-rt, N_2_; (vi) NCS (**9a**) or NIS (**9c**), MeCN, reflux; NBS (**9b**), MeCN, r.t.; (vii) for **9d**, MeONa, MeOH, reflux, N_2_; for **9e**, EtONa, EtOH, reflux, N_2_; (viii) aq LiOH, EtOH, r.t.; (ix) aq NaOH, MeOH, r.t.

The synthetic route to target compounds **1a**–**1f** was shown in Scheme 1. Ester **2a** was treated with 80% aqueous hydrazine hydrate in methanol at room temperature to smoothly give acyl hydrazide **3a**. An efficient one-pot, three-component synthetic approach was employed to construct the desired 1,2,4-triazole core, which involved the initial condensation of **3a** and *N*,*N*-dimethylformamide dimethyl acetal (DMFDMA) in acetonitrile at 50 °C in an open vessel to produce an adduct that in turn reacted with amine **4a** added subsequently in refluxing acetic acid to furnish 1,2,4-triazole **5a** via ring closing [20]. Dihydroxylation of olefin **5a** by treatment of **5a** with osmium tetroxide and *N*-methylmorpholine *N*-oxide (NMMO) in THF/H_2_O (4/1) at room temperature afforded the corresponding diol, which, after isolation and purification, was further treated with sodium periodate in THF/H_2_O (4/1) at room temperature to yield aldehyde **6a** [21]. Pinnick oxidation of aldehyde **6a** with NaClO_2_ in the presence of NaH_2_PO_4_ and 2-methyl-2-butene in *t*-BuOH/H_2_O (4/1) at room temperature cleanly produced the corresponding carboxylic acid **7a** [22]. Esterification of **7a** with methanol in the presence of 1-ethyl-3-(3-dimethylaminopropyl)carbodiimide hydrochloride (EDCI) and 4-dimethylaminopyridine (DMAP) in dichloromethane at room temperature smoothly produced ester **8a**. However, an initial attempt to achieve this esterification with 1,3-dicyclohexylcarbodiimide (DCC) in replacement of EDCI was unsatisfactory because an appreciable amount of intermediate resulting from the adduction of **7a** and DCC was observed even in refluxing tetrahydrofuran, which eventually resulted in a quite low yield of **8a**. Halogenation at 5-position of 1,2,4-triazole ring in **8a** was achieved by treatment of **8a** with *N*-halosuccinimides (NCS, NBS and NIS) in acetonitrile at room temperature (NBS) or at reflux (NCS and NIS), leading to the formation of **9a**–**9c**. Aromatic nucleophilic substitution of the Br atom in **9b** by treatment of **9b** with MeONa and EtONa in corresponding alcohols at reflux successfully produced **9d** and **9e**, respectively, albeit in quite low yields (14% for **9d** and 13% for **9e**). It should be noted that methyl/ethyl transesterification occurred in the case of **9e**. Alkaline hydrolysis of esters **9a**–**9e** with aqueous LiOH in methanol at room temperature afforded corresponding carboxylic acids **10a**–**10e**. Finally, acids **7a** and **10a**–**10e** were converted to sodium salts thereof **1a**–**1f** with aqueous NaOH in methanol for the sake of improving the solubility in in vitro URAT1 inhibitory assay.

**Scheme 2:**
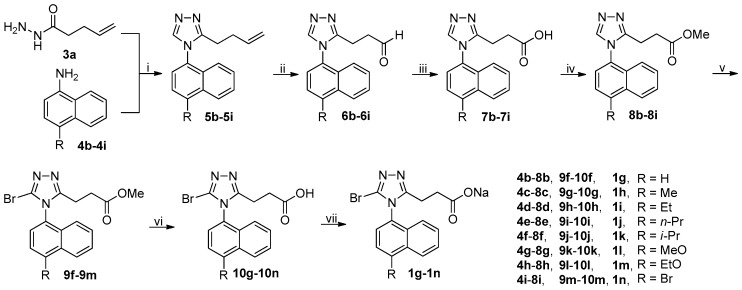
Synthetic route to **1g**–**1n**. *Reagents and conditions*: (i) DMFDMA, **3a**, MeCN, 50 °C, open vessel; (b) then **4b**–**4i**, AcOH, reflux; (ii) (a) NMMO, OsO_4_, THF/H_2_O (4/1), r.t.; (b) NaIO_4_, THF/H_2_O (4/1), r.t.; (iii) NaClO_2_, NaH_2_PO_4_, 2-methyl-2-butene, *t*-BuOH/H_2_O (4/1), 0 °C-rt; (iv) MeOH, EDCI, DMAP, CH_2_Cl_2_, 0 °C-rt, N_2_; (v) NBS, MeCN, r.t. (for **8b**–**8h**) or 60 °C (for **8i**); (vi) aq LiOH, EtOH, r.t.; (vii) aq NaOH, MeOH, r.t.

The synthetic route to target compounds **1g**–**1n** was summarized in Scheme 2. Following the identical procedure used for the synthesis of **1c** starting from **3a** and **4a**, target compounds **1g**–**1n** were successfully prepared from **3a** and **4b**–**4i**. It should be noted that brominations of **8g** and **8h** with NBS at room temperature led to the formation of large amounts of byproducts (byproducts/**9k** or **9l** = 55/45) that were preliminarily identified by ^1^H-NMR to result from the bromination at the naphthalene ring instead of the desired 5-position of 1,2,4-triazole ring. This phenomenon is consistent with the theoretical prediction that the naphthalene ring activated by strongly electron-donating alkoxy groups readily undergoes electrophilic substitution; on the contrary, the bromination of **8i** needed elevated temperature (60 °C) to reach a reasonable reaction rate due to the presence of a weakly electron-withdrawing Br atom at 4-position of the naphthalene ring. In an unsuccessful attempt, it was experimentally found that a counterpart of **8i** with R being NO_2_, a strongly electron-withdrawing group, could not even be brominated with NBS even in refluxing acetonitrile.

**Scheme 3:**
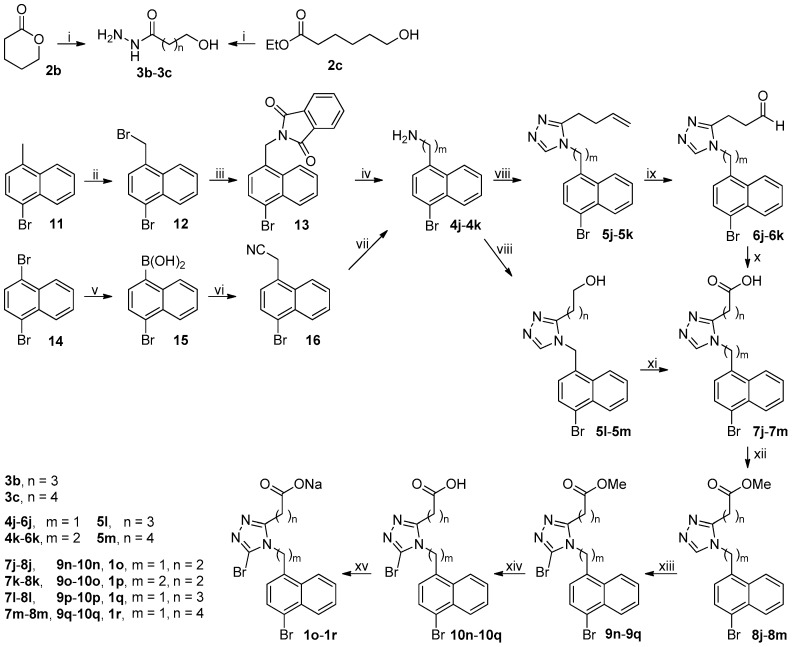
Synthetic route to **1o**–**1r**. *Reagents and conditions*: (i) 80% N_2_H_4_·H_2_O, MeOH, r.t. (**2b**) or reflux (**2c**); (ii) NBS, BPO, *n*-hexane, reflux, N_2_; (iii) potassium phthalimide, DMF, 100°C, N_2_; (iv) 80% N_2_H_4_·H_2_O, EtOH, reflux; (v) (a) *n*-BuLi (1.0 eq), THF, −78 °C; (b) B(*i*-PrO)_3_, −78 °C-rt; (vi) CNCH_2_NH_2_·HCl, NaNO_2_, PhMe/H_2_O (20/1), 50 °C; (vii) LiAlH_4_, THF, 0 °C-rt, N_2_; (viii) (a) DMFDMA, **3a**–**3c**, MeCN, 50 °C, open vessel; (b) **4j**–**4k**, AcOH, reflux; (ix) (a) NMMO, OsO_4_, THF/H_2_O (4/1), r.t.; (b) NaIO_4_, THF/H_2_O (4/1), r.t.; (x) NaClO_2_, NaH_2_PO_4_, 2-methyl-2-butene, *t*-BuOH/H_2_O (4/1), 0 °C-rt; (xi) NaClO_2_, TEMPO, NaClO, MeCN, phosphate buffer (pH = 6.7), 35 °C; (xii) MeOH, EDCI, DMAP, CH_2_Cl_2_, 0 °C-rt, N_2_; (xiii) NBS, MeCN, r.t.; (xiv) aq LiOH, EtOH, r.t.; (xv) aq NaOH, MeOH, r.t.

The synthetic route to target compounds **1o**–**1r** was depicted in Scheme 3. Esters **2b** and **2c** were hydrazinolyzed to acyl hydrazides **3b** and **3c**, respectively, following the procedure used for the synthesis of **3a** from **2a** except that the hydrazinolysis of **2c** needed to be carried out at reflux. Compound **11** was treated with NBS in the presence of benzoyl peroxide (BPO) in refluxing *n*-hexane to furnish **12**, which was in turn transformed to corresponding amine **4j** via Gabriel synthesis involving the reaction of **12** and potassium naphthalimide in DMF at 100 °C to give **13** and subsequent hydrazinolysis of **13** with 80% aqueous hydrazine hydrate in refluxing ethanol to yield **4j** [23]. Transformation of bisbromide **14** to mono-boronic acid **15** was achieved by treatment of **14** with 1 eq of *n*-BuLi at −78 °C followed by quenching with triisopropyl borate. Aryl boronic acid **15** was coupled with aminoacetonitrile to afford aryl acetonitrile **16** according to an efficient deaminative coupling approach which involved the reaction of **15** and aminoacetonitrile hydrochloride in the presence of sodium nitrite in toluene/H_2_O (20/1) at 50 °C [24]. An initial attempt to construct aryl acetonitrile **16** by Suzuki coupling, which involved the reaction of **15** and chloroacetonitrile under a well-established Pd-catalyzed condition [25,26], was unsuccessful in that a complex mixture of unidentified products was formed presumably due to the competitive reactions resulting from the presence of two cross-coupling acceptors (aryl bromide **15** and chloroacetonitrile) in this case. Reduction of aryl acetonitrile **16** with lithium aluminum hydride in dried THF at room temperature smoothly afforded desired amine **4k**. Amines **4j**–**4k** and hydrazide **3a** were transformed to **7j**–**7k** following the same procedure used for the synthesis of **7a** from **4a** and **3a**. On the other hand, amine **4j** and hydrazides **3b**–**3c** were subjected to the same procedure used for the synthesis of **5a** from **4a** and **3a** to produce **5l**–**5m**. TEMPO/NaClO-catalyzed oxidation of **5l**–**5m** with sodium chlorite in acetonitrile/phosphate buffer (pH = 6.7) at 35 °C smoothly produced corresponding carboxylic acids **7l**–**7m**, respectively [27]. Carboxylic acids **7j**–**7m** were transformed to target compounds **1o**–**1r** following the procedure for the synthesis of **1c** from **7a**.

**Scheme 4:**
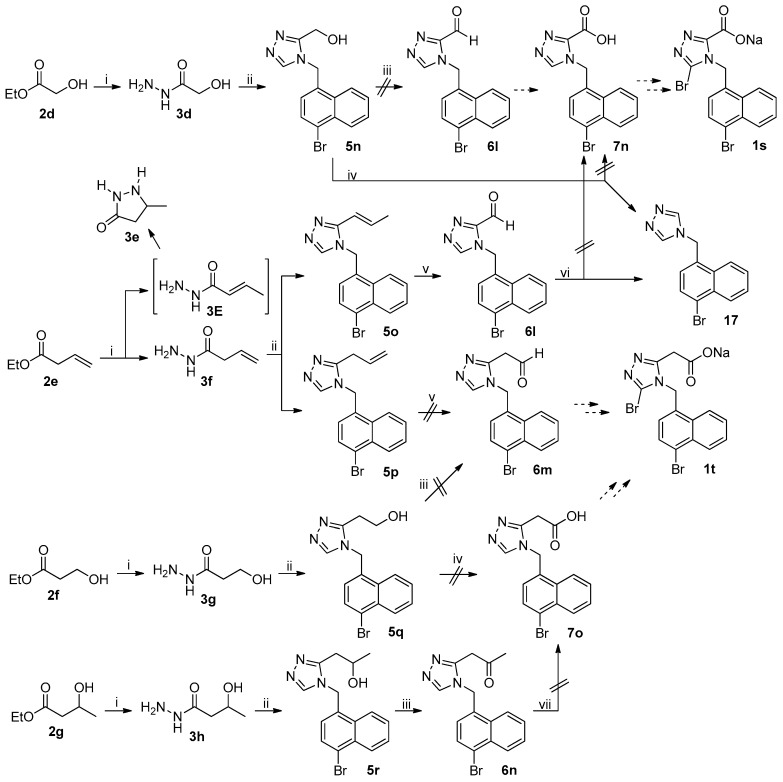
Unsuccessful synthetic route to **1s**–**1t**. *Reagents and conditions*: (i) 80% N_2_H_4_·H_2_O, MeOH, r.t.; (ii) (a) DMFDMA, **3d**–**3h**, MeCN, 50 °C, open vessel; (b) **4j**, AcOH, reflux; (iii) (a) (COCl)_2_, DMSO, **5n**, **5q**, or **5r**, CH_2_Cl_2_, −60 °C; (b) Et_3_N, −60 °C to r.t.; (iv) NaClO_2_, TEMPO, NaClO, MeCN, phosphate buffer (pH = 6.7), 35 °C; (v) (a) NMMO, OsO_4_, THF/H_2_O (4/1), r.t.; (b) NaIO_4_, THF/H_2_O (4/1), r.t.; (vi) NaClO_2_, NaH_2_PO_4_, 2-methyl-2-butene, *t*-BuOH/H_2_O (4/1), 0 °C-rt; (vii) I_2_ or Br_2_, NaOH, 1,4-dioxane/H_2_O, rt-reflux.

The unsuccessful synthetic routes to anticipated target compounds **1s** and **1t** were depicted in Scheme 4. Ester **2d** was transformed to **5n** according to the procedure used for the synthesis of **5l**–**5m** from **2b**–**2c** described above. Swern oxidation of triazolylmethanol **5n** to corresponding triazolylaldehyde **6l** involving the treatment of **5n** with (COCl)_2_/DMSO/Et_3_N [28] was unsuccessful in that a complex mixture of unidentified products was formed. Aldehyde **6l** was initially expected to be transformed to anticipated target compound **1s** by the procedure used for the synthesis of **1c** from **6a**. Direct oxidation of primary alcohol **5n** to corresponding carboxylic acid **7n** with TEMPO/NaClO-catalyzed oxidation, aforementioned, to avoid the involvement of aldehyde **6l** was also unsuccessful because **17** was isolated as the major product that clearly resulted from the decarboxylation of **7n**, strongly indicating that **7n** was probably an unstable compound. Hydrazinolysis of **2e** under the reaction conditions described above led to the formation of **3e** and **3f** in a ratio of approximately 1/9; the isolation of **3e** unambiguously suggested the formation of **3E** during the hydrazinolysis of **2e**, an isomer of **3f** resulting from the C=C double bond migration [29]. 1,2,4-Triazole formation from **3f** and **4j** under the reaction conditions as described above led to the formation of **5o** and **5p** in a ratio of approximately 1/5, suggesting that the C=C double bond migration also occurred to a notable extent in this reaction. Transformation of olefin **5o** to aldehyde **6l** according to the procedure used for the transformation of **5a** to **6a** described above was indeed successful, but further oxidation of aldehyde **6l** to carboxylic acid **7n** by Pinnick oxidation, aforementioned, failed due to the isolation of decarboxylated product **17** once again, further confirming that triazolylcarboxylic acid **7n** was not a stable compound (but the corresponding triazolylaldehyde **6l** was). On the other hand, the cleavage of olefin **5p** with anticipation to furnish acetaldehyde **6m** as described above failed due to the formation of a complex mixture of unidentified products; the latter was initially expected to be used for the synthesis of anticipated target compounds **1t**. Ethyl alcohol **5q** was prepared from **2f** by the procedure used for the synthesis of **5l**–**5m** from **2b**–**2c**. Oxidation of **5q** to acetaldehyde **6m** by Swern oxidation described above also failed, strongly suggesting that triazolylacetaldehyde **6m** was unstable. Direct oxidation of **5q** to corresponding acetic acid **7o** also failed, leading to a complex mixture of unidentified products. Secondary alcohol **5r** was prepared from **2g** following the procedure described above. Swern oxidation of **5r** under the reaction conditions described above successfully furnished corresponding acetone **6n**, but further transformation of methyl ketone **6n** to acetic acid **7o** by haloform reaction [30] also failed, indicating that triazolylacetic acid **7o** was probably unstable.

**Scheme 5:**
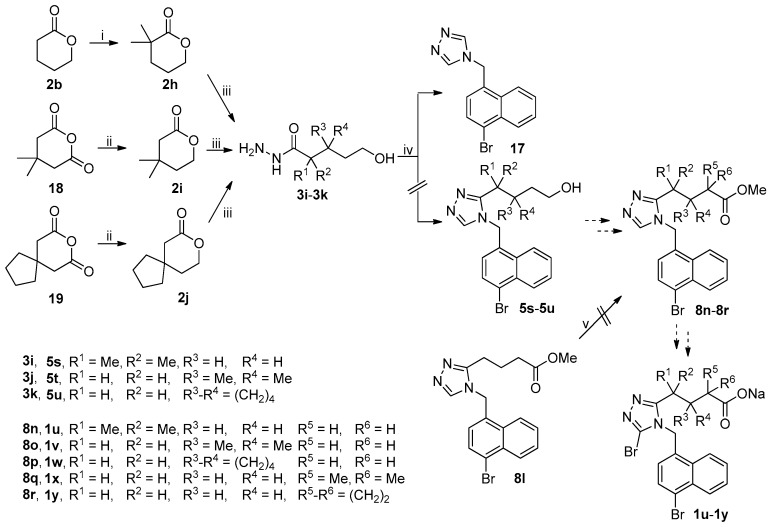
Unsuccessful synthetic route to **1u**–**1y**. *Reagents and conditions*: (i) LDA, THF, −78 °C, then MeI, DMPU, −78 °C-rt (two cycles); (ii) NaBH_4_, THF, 0 °C-rt, then 6 M HCl; (iii) 80% N_2_H_4_·H_2_O, MeOH, r.t.; (iv) (a) DMFDMA, **3i**–**3k**, MeCN, 50 °C, open vessel; (b) **4j**, AcOH, reflux; (v) for **8q**: LDA, THF, −78 °C, then MeI, DMPU, −78 °C-rt (two cycles); for **8r**: LDA, THF, −78 °C, then 1,3,2-dioxathiolane-2,2-dioxide, DMPU, −78 °C-rt.

The unsuccessful synthetic routes to anticipated target compounds **1u**–**1y** were depicted in Scheme 5. Ester **2b** was treated with lithium diisopropylamide (LDA) generated in situ from *n*-BuLi and diisopropylamine in dried THF at −78 °C and then quenched by methyl iodide and 1,3-dimethyltetrahydropyrimidin-2(1*H*)-one (DMPU) at −78 °C to room temperature and this process was repeated twice to give rise to **2h** with the *gem*-dimethyl group at the α-position of carboxylate [31,32]. Reduction of anhydrides **18** and **19** with sodium borohydride in THF at 0 °C to room temperature followed by treatment with 6 M hydrochloric acid afforded esters **2i** and **2j**, respectively [33]. Hydrazinolysis of **2h**–**2j** under the reaction conditions described above gave acyl hydrazides **3i**–**3k**. 1,2,4-Triazole ring formation with acyl hydrazides **3i**–**3k** and amine **4j** under the reaction conditions described above failed because all three reactions uniformly yielded **17** as the major product instead of the desired **5s**–**5u** which were initially expected to be employed for the synthesis of anticipated target compounds **1u**–**1w**. Attempts to introduce a *gem*-dimethyl group via the approach for the synthesis of **2h** from **2b** described above and a cyclopropyl group via sequential deprotonation with LDA and quenching with 1,3,2-dioxathiolane-2,2-dioxide [34] at the α-position of carboxylate in **8l** to prepare **8q** and **8r**, respectively, were unsuccessful because a complex mixture of unidentified products was formed in both cases. Esters **8q** and **8r** were initially expected to be used for the synthesis of the anticipated target compounds **1x** and **1y**, respectively.

### 2.2. In Vitro URAT1 Inhibitory Activity

Table 1 summarized the results for the in vitro inhibitory activity of **1a**–**1r** as well as lesinurad as a positive control against human URAT1. Replacement of the S atom in lesinurad with CH_2_ led to the increase of in vitro URAT1 inhibitory activity by three fold (**1c** vs. lesinurad), suggesting that the S atom was probably not indispensable and its replacement with CH_2_ could even increase the URAT1 inhibitory activity. In the series of designed compounds **1a**–**1r** in the present study, the SAR exploration commenced with the screening of the substituents at the 5-position of the 1,2,4-triazole ring (**1a**–**1f**), and it turned out that H and OMe led to the complete loss of bioactivity, while the three halogen atoms, Cl, Br, and I, as well as OEt, maintained the IC_50_ at the same order of magnitude, with Br being the most favorable substituent. In the second round of SAR exploration, we turned to screen the substituents at the 4-position of the naphthalene ring while fixing the Br atom at 5-position of the triazole ring (**1c** and **1g**–**1n**). It was very obvious that the bioactivity essentially increased as the steric volume of the alkyl substituent increased (**1c** and **1h**–**1k**), with *i*-Pr being the most favorable one. OMe was detrimental to the bioactivity as demonstrated by the dramatic drop of the bioactivity of **1l**. The most potent bioactivity in this round was observed from **1n**, with Br emerging as the optimal substituent at this position. With Br being discovered to be the optimal substituents for both positions aforementioned, we went on to explore the lengths of the two linkers connecting the naphthalene and triazole rings as well as the triazole and carboxylate functionality, respectively (**1n**–**1p** for “m”; **1o**, **1q** and **1r** for “n”). The linker length “m” was screened first (**1n**–**1p**), and it was very clear that the bioactivity first increased slightly and then decreased dramatically when the “m” increased from 0 to 2, with m = 1 being the most favorable linker length. By fixing m = 1, we subsequently screened the linker length “n” (**1o**, **1q** and **1r**). A similar trend to that for “m” was observed, and when *n* = 3 the bioactivity was most potent, with **1q** emerging as the most potent URAT1 inhibitor among all the designed target compounds **1a**–**1r**, which was 31-fold more potent than lesinurad.

Actually, as shown in Scheme 4 and Scheme 5, besides the compounds **1a**–**1r**, we originally designed **1s**–**1t** and **1u**–**1y** with anticipation to further explore the SAR of the linker length “n” and the substituents at the side chain at the 3-position of triazole in **1q**, respectively. However, as discussed above, none of these designed compounds were synthetically accessible although considerable efforts have been made.

## 3. Experimental Section

### 3.1. General

Melting points were measured with an RY-2 microscopic melting point apparatus and are uncorrected. ^1^H-NMR and ^13^C-NMR spectra were recorded on a Bruker AV400 NMR spectrometer (Bruker BioSpin AG, Faellanden, Switzerland), using DMSO-*d*_6_, CDCl_3_ or MeOH-*d*_4_ as solvent and known chemical shifts of residual proton signals of deuterated solvents (for ^1^H-NMR) or carbon signals of deuterated solvents (for ^13^C-NMR) as the internal standard. High-resolution mass spectra (HRMS) were determined with an Agilent Q-TOF 6510 mass spectrometer (Agilent Technologies, Santa Clara, CA, USA) using the direct injection method, and electrospray ionization (ESI) was used as an ionization technique in negative mode (ESI−).

Esters **2a**–**2g** and amines **4a**–**4i** were commercially available. All the dried solvents were prepared by standard methods. 

### 3.2. Chemistry

#### 3.2.1. Synthesis of 2,2-Dimethyl-δ-valerolactone (**2h**)

To a magnetically stirred solution of diisopropylamine (23.27 g, 230 mmol) in dried THF (250 mL) cooled at −78 °C under N_2_ was added dropwise 1.6 M *n*-BuLi in *n*-hexane (131 mL, 210 mmol) via syringe. After addition, the resulting mixture was stirred at this temperature for 10 min, followed by dropwise addition of δ-valerolactone **2b** (10.01 g, 100 mmol). The stirring was continued at this temperature for another 0.5 h, followed by successive additions of MeI (28.39 g, 200 mmol) and DMPU (14.10 g, 110 mmol) in a dropwise manner via syringe. After addition, the reaction mixture was stirred at room temperature overnight.

The reaction mixture was concentrated on a rotary evaporator to about 100 mL and then poured into ice-water (500 mL). The resulting aqueous mixture was extracted with CH_2_Cl_2_ (100 mL × 3), and the combined extracts were washed successively with 1 M hydrochloric acid (100 mL × 3) and 5% brine (100 mL), dried over anhydrous Na_2_SO_4_, and evaporated on a rotary evaporator to afford a residue, which was purified by column chromatography to yield a colorless oil. The colorless oil was subjected to the identical procedure described above once again to yield **2h** after column chromatography. Colorless oil, 9.10 g (71%). ^1^H-NMR (DMSO-*d*_6_, 400 MHz) δ: 4.27 (t, *J* = 5.8 Hz, 2H), 1.78–1.84 (m, 2H), 1.68–1.71 (m, 2H), 1.17 (s, 6H). The ^1^H-NMR data were in good agreement with those reported [35].

#### 3.2.2. General Procedure for the Synthesis of Lactones **2i** and **2j**

To a stirred suspension of NaBH_4_ (5.67 g, 150 mmol) in dried THF (50 mL) cooled in an ice-water bath was added dropwise a solution of **18** or **19** (100 mmol) in dried THF (50 mL). The resulting mixture was stirred at room temperature for 5 h and then re-cooled in an ice-water bath, followed by addition of 6 M hydrochloric acid (50 mL). The mixture thus obtained was stirred for another 5 min and poured into ice-water (300 mL). The resulting mixture was extracted with CH_2_Cl_2_ (100 mL × 3), and the combined extracts were washed with 5% brine (100 mL), dried over anhydrous Na_2_SO_4_, and evaporated on a rotary evaporator to afford a residue, which was purified by column chromatography to yield **2i** or **2j**.

*3,3-Dimethyl-δ-valerolactone* (**2i**): Colorless oil; 9.36 g (73%). ^1^H-NMR (DMSO-*d*_6_, 400 MHz) δ: 4.28 (t, *J* = 6.2 Hz, 2H), 2.27 (s, 2H), 1.62 (t, *J* = 6.0 Hz, 2H), 0.99 (s, 6H). The ^1^H-NMR data were in good agreement with those reported [36].

*8-Oxaspiro[4,5]decan-7-one* (**2j**): Colorless oil; 11.41 g (74%). ^1^H-NMR (DMSO-*d*_6_, 400 MHz) δ: 4.28 (t, *J* = 6.0 Hz, 2H), 2.37 (s, 2H), 1.70 (t, *J* = 6.2 Hz, 2H), 1.45–1.62 (m, 4H), 1.37–1.42 (m, 2H).

#### 3.2.3. General Procedure for the Synthesis of Acyl Hydrazides **3a**–**3k**

To a stirred solution of esters **2a**–**2j** (70 mmol) in MeOH (30 mL) cooled in an ice-water bath was added dropwise 80% aqueous hydrazine hydrate (6.26 g, 100 mmol). The resulting solution was stirred at room temperature (**2a**–**2b** or **2d**–**2j**), or reflux (**2c**), until the completion of reaction as indicated by TLC analysis (typically within 5 h).

The reaction mixture was evaporated on a rotary evaporator to give a residue, which was purified by column chromatography through a short silica gel column to yield **3a**–**3k** after trituration with *n*-hexane if possible.

*4-Pentenoyl hydrazide* (**3a**): White solid; 6.47 g (81%); m.p. 44.5–45.5 °C (literature value, 45 °C [37]). ^1^H-NMR (DMSO-*d*_6_, 400 MHz) δ: 8.92 (brs, 1H), 5.72–5.82 (m, 1H), 4.98–5.03 (m, 1H), 4.92–4.95 (m, 1H), 4.13 (brs, 2H), 2.20–2.25 (m, 2H), 2.08 (t, *J* = 7.6 Hz, 2H).

*5-Hydroxypentanoyl hydrazide* (**3b**): White solid; 7.59 g (82%); m.p. 108–109.5 °C (literature value, 107–108 °C [38]). ^1^H-NMR (DMSO-*d*_6_, 400 MHz) δ: 8.89 (brs, 1H), 4.34 (brs, 1H), 4.12 (brs, 2H), 3.35 (t, *J* = 6.4 Hz, 2H), 1.99 (t, *J* = 7.4 Hz, 2H), 1.45–1.53 (m, 2H), 1.33–1.40 (m, 2H).

*6-Hydroxyhexanoyl hydrazide* (**3c**): White solid; 8.39 g (82%); m.p. 116.5–118 °C (literature value, 114.5–116 °C [39]). ^1^H-NMR (DMSO-*d*_6_, 400 MHz) δ: 8.88 (brs, 1H), 4.31 (t, *J* = 5.0 Hz, 1H), 4.11 (brs, 2H), 3.35 (q, *J* = 6.0 Hz, 2H), 1.98 (t, *J* = 7.6 Hz, 2H), 1.42–1.50 (m, 2H), 1.35–1.40 (m, 2H), 1.19–1.26 (m, 2H).

*2-Hydroxyacetyl hydrazide* (**3d**): White solid; 5.36 g (85%); m.p. 91.5–93 °C (literature value, 93 °C [40]). ^1^H-NMR (DMSO-*d*_6_, 400 MHz) δ: 8.81 (brs, 1H), 5.28 (brs, 1H), 4.20 (brs, 2H), 3.82 (s, 2H).

*5-Methylpyrazolidin-3-one* (**3e**): Colorless thick oil; 0.56 g (8%); ^1^H-NMR (DMSO-*d*_6_, 400 MHz) δ: 8.89 (brs, 1H), 5.06 (brs, 1H), 3.46-3.51 (m, 1H), 2.29 (dd, *J* = 7.0 Hz and 15.4 Hz, 1H), 1.91 (dd, *J* = 8.2 Hz and 15.8 Hz, 1H), 1.09 (d, *J* = 6.4 Hz, 3H). The ^1^H-NMR data were in good agreement with those reported [29].

*3-Butenoyl hydrazide* (**3f**): White solid; 5.19 g (74%); m.p. 47.5–48.5 °C. ^1^H-NMR (DMSO-*d*_6_, 400 MHz) δ: 8.97 (brs, 1H), 5.79–5.89 (m, 1H), 5.03–5.10 (m, 2H), 4.15 (brs, 2H), 2.81 (d, *J* = 6.8 Hz, 2H). The ^1^H-NMR data were in good agreement with those reported [41].

*3-Hydroxypropionyl hydrazide* (**3g**): White solid; 5.76 g (79%); m.p. 102–103.5 °C (literature value, 103–104 °C [42]). ^1^H-NMR (DMSO-*d*_6_, 400 MHz) δ: 8.93 (brs, 1H), 4.57 (brs, 1H), 4.15 (brs, 2H), 3.58 (t, *J* = 6.6 Hz, 2H), 2.16 (t, *J* = 6.6 Hz, 2H).

*3-Hydroxybutanoyl hydrazide* (**3h**): White solid; 6.86 g (83%); m.p. 124–126 °C (literature value, 126–128 °C [43]). ^1^H-NMR (DMSO-*d*_6_, 400 MHz) δ: 8.90 (brs, 1H), 4.58 (brs, 1H), 4.12 (brs, 2H), 3.91–3.98 (m, 1H), 2.14 (dd, *J* = 7.0 Hz and 13.8 Hz, 1H), 2.03 (dd, *J* = 6.0 Hz and 13.6 Hz, 1H), 1.03 (d, *J* = 6.0 Hz, 3H).

*5-Hydroxy-2,2-dimethylpentanoyl hydrazide* (**3i**): Colorless oil; 9.76 g (87%). ^1^H-NMR (DMSO-*d*_6_, 400 MHz) δ: 8.71 (brs, 1H), 4.32 (t, *J* = 5.2 Hz, 1H), 4.13 (brs, 2H), 3.28–3.33 (m, 2H), 1.37–1.41 (m, 2H), 1.23–1.33 (m, 2H), 1.03 (s, 6H).

*5-Hydroxy-3,3-dimethylpentanoyl hydrazide* (**3j**): White solid; 9.20 g (82%); m.p. 57.5–59 °C. ^1^H-NMR (DMSO-*d*_6_, 400 MHz) δ: 8.87 (brs, 1H), 4.33 (brs, 1H), 4.16 (brs, 2H), 3.44–3.47 (m, 2H), 1.91 (s, 2H), 1.44 (t, *J* = 7.4 Hz, 2H), 0.91 (s, 6H).

*2-(1-(2-Hydroxyethyl)cyclopentyl)acetyl hydrazide* (**3k**): Colorless oil; 11.21 g (86%). ^1^H-NMR (DMSO-*d*_6_, 400 MHz) δ: 8.90 (brs, 1H), 4.38 (brs, 1H), 4.16 (brs, 2H), 3.47 (t, *J* = 7.2 Hz, 2H), 1.99 (s, 2H), 1.49–1.57 (m, 8H), 1.30–1.37 (m, 2H).

#### 3.2.4. Synthesis of (4-Bromonaphth-1-yl)methylamine **4j**

A suspension of **11** (35.37 g, 160 mmol), BPO (0.78 g, 3.2 mmol), and NBS (34.17 g, 192 mmol) in *n*-hexane (400 mL) was refluxed under N_2_ until the completion of reaction as indicated by TLC analysis (typically 36 h; once the reaction commenced, 0.78 g of BPO was added every 8 h until the reaction completed). The reaction mixture was cooled to room temperature while stirring, and the precipitates were collected via vacuum filtration. The precipitates were triturated successively with saturated aqueous NaHCO_3_ (500 mL × 2), water (800 mL × 2), and *n*-hexane (800 mL) to give rise to (4-bromonaphth-1-yl)methyl bromide **12**. White solid; 35.04 g (73%); m.p. 104.5–106 °C (literature value, 102–104 °C [44]). ^1^H-NMR (DMSO-*d*_6_, 400 MHz) δ: 8.20–8.26 (m, 2H), 7.85 (d, *J* = 7.6 Hz, 1H), 7.71–7.77 (m, 2H), 7.62 (d, *J* = 7.6 Hz, 1H), 5.21 (s, 2H).

A mixture of **12** (33.00 g, 110 mmol) and potassium phthalimide (20.37 g, 110 mmol) in DMF (200 mL) was stirred at 100 °C under N_2_ until the completion of reaction as indicated by TLC analysis (typically within 12 h). On cooling to room temperature, the reaction mixture was poured into ice-water (600 mL), and the aqueous mixture thus obtained was extracted with CH_2_Cl_2_ (150 mL × 3). The combined extracts were washed with 5% brine (100 mL × 5), dried over anhydrous Na_2_SO_4_, and evaporated on a rotary evaporator to afford a residue, which was recrystallized from ethanol to produce *N*-((4-bromonaphth-1-yl)methyl)phthalimide **13**. White solid; 36.66 g (91%); m.p. 168.5–170 °C. ^1^H-NMR (DMSO-*d*_6_, 400 MHz) δ: 8.30–8.33 (m, 1H), 8.17–8.21 (m, 1H), 7.83–7.92 (m, 4H), 7.80 (d, *J* = 8.0 Hz, 1H), 7.69–7.74 (m, 2H), 7.32 (d, *J* = 7.6 Hz, 1H), 5.23 (s, 2H).

A mixture of **13** (32.96 g, 90 mmol) and 80% aqueous hydrazine hydrate (11.26 g, 180 mmol) in ethanol (600 mL) was refluxed until the completion of reaction as indicated by TLC analysis (typically within 12 h), when a white slurry was formed. On slight cooling, 1 M aqueous NaOH (300 mL) was added to the reaction mixture, which turned to a clear solution and was concentrated on a rotary evaporator to half its original volume. The residue was poured into ice-water (350 mL). The aqueous mixture thus obtained was extracted with CH_2_Cl_2_ (100 mL × 3). The combined extracts were washed successively with 5% aqueous NaOH (100 mL × 2) and 5% brine (100 mL), dried over anhydrous Na_2_SO_4_, and evaporated on a rotary evaporator to afford a residue, which was purified by column chromatography through a short silica gel column to produce **4j**. Colorless oil; 18.27 g (86%). ^1^H-NMR (DMSO-*d*_6_, 400 MHz) δ: 8.15–8.18 (m, 2H), 7.83 (d, *J* = 7.6 Hz, 1H), 7.61–7.70 (m, 2H), 7.49 (d, *J* = 7.6 Hz, 1H), 4.17 (s, 2H), 1.94 (brs, 2H).

#### 3.2.5. Synthesis of 2-(4-Bromonaphth-1-yl)ethylamine **4k**

To a magnetically stirred solution of 1,4-dibromonaphthalene **14** (57.19 g, 200 mmol) in dried THF (600 mL) cooled at −78 °C under N_2_ was added dropwise 1.6 M *n*-BuLi in *n*-hexane (125 mL, 200 mmol) via syringe. After addition, the resulting mixture was stirred at this temperature for another 0.5 h, followed by addition of B(*i*-PrO)_3_ (75.23 g, 400 mmol) in a dropwise manner via syringe. The reaction mixture was slowly warmed to room temperature and stirred at room temperature for another 1 h. The reaction mixture was slowly poured into ice-water (600 mL) with concentrated hydrochloric acid (10 mL) while stirring. The precipitates formed were collected via vacuum filtration, washed with cooled water, and triturated with EtOAc/*n*-hexane to yield 4-bromonaphthalene-1-boronic acid **15**. White solid; 46.16 g (92%). A varying amount of boronic anhydride was found to exist in the sample of **15** and therefore **15** was directly used in the next step without further structural characterization.

A mixture of **15** (45.16 g, 180 mmol), aminoacetonitrile hydrochloride (33.31 g, 360 mmol) and NaNO_2_ (31.05 g, 450 mmol) in toluene (600 mL)/water (30 mL) was stirred at 50 °C until the completion of reaction as indicated by TLC analysis (typically within 12 h). On cooling to room temperature, the reaction mixture was poured into ice-water (300 mL) and the organic phase was separated. The aqueous phase was back-extracted with toluene (100 mL). The combined extracts were washed with 5% brine, dried over anhydrous Na_2_SO_4_, and evaporated on a rotary evaporator to afford a residue, which was purified by column chromatography to produce 2-(4-bromonaphth-1-yl)acetonitrile **16**. White solid; 35.88 g (81%); m.p. 74.5–75.5 °C. ^1^H-NMR (DMSO-*d*_6_, 400 MHz) δ: 8.21–8.24 (m, 1H), 8.08–8.11 (m, 1H), 7.92 (d, *J* = 7.6 Hz, 1H), 7.74–7.79 (m, 2H), 7.53 (d, *J* = 7.6 Hz, 1H), 4.50 (s, 2H).

To a stirred solution of **16** (34.45 g, 140 mmol) in dried THF (300 mL) cooled in an ice-water bath was added portionwise LiAlH_4_ (10.63 g, 280 mmol). The resulting mixture was stirred at room temperature under N_2_ until the completion of reaction as indicated by TLC analysis (typically within 12 h). An appropriate amount of water was carefully added to the stirred reaction mixture to decompose the excess LiAlH_4_, and the mixture thus obtained was filtered off through celite. The filtrate was poured into ice-water (600 mL), and the resulting mixture was extracted with CH_2_Cl_2_ (200 mL × 3). The combined extracts were washed with 5% brine (100 mL), dried over anhydrous Na_2_SO_4_, and evaporated on a rotary evaporator to afford a residue, which was purified by column chromatography through a short silica gel column to produce **4k**. Colorless oil; 25.56 g (73%). ^1^H-NMR (DMSO-*d*_6_, 400 MHz) δ: 8.15–8.19 (m, 2H), 7.79 (d, *J* = 7.6 Hz, 1H), 7.64–7.69 (m, 2H), 7.29 (d, *J* = 7.6 Hz, 1H), 3.11 (t, *J* = 7.4 Hz, 2H), 2.84 (t, *J* = 7.4 Hz, 2H), 1.73 (brs, 2H).

#### 3.2.6. General Procedure for the Synthesis of **5a**–**5r** and **17**

A mixture of **3a**–**3d** or **3f**–**3k** (75 mmol) and DMFDMA (8.94 g, 75 mmol) in MeCN (100 mL) was stirred at 50 °C in an open vessel in a well-ventilated hood until the completion of reaction as indicated by TLC analysis (typically within 2 h). The reaction mixture was evaporated on a rotary evaporator to almost dryness and the residue thus obtained was dissolved in glacial acetic acid (100 mL), followed by addition of **4a**–**4k** (75 mmol). The resulting mixture was refluxed until the completion of reaction as indicated by TLC analysis (typically within 12 h).

On slight cooling, the reaction mixture was concentrated on a rotary evaporator to approximately 50 mL and poured into ice-water (600 mL) while stirring, and the resulting mixture was extracted with CH_2_Cl_2_ (200 mL × 3). The combined extracts were washed successively with 1 M hydrochloric acid (100 mL), saturated aqueous NaHCO_3_ (until the aqueous pH > 7 persistently), and 5% brine (100 mL), dried over anhydrous Na_2_SO_4_, and evaporated on a rotary evaporator to afford a residue, which was purified by column chromatography to produce **5a**–**5r** or **17** after trituration with EtOAc/*n*-hexane if possible.

*3-(3-Buten-1-yl)-4-(4-cyclopropylnaphth-1-yl)-4H-1,2,4-triazole* (**5a**): Colorless oil; 16.93 g (78%). ^1^H-NMR (DMSO-*d*_6_, 400 MHz) δ: 8.68 (s, 1H), 8.55 (d, *J* = 8.4 Hz, 1H), 7.69–7.74 (m, 1H), 7.61–7.65 (m, 1H), 7.55 (dd, *J* = 3.4 Hz and 7.4 Hz, 1H), 7.39 (d, *J* = 7.6 Hz, 1H), 7.12 (d, *J* = 8.4 Hz, 1H), 5.61–5.71 (m, 1H), 4.83–4.88 (m, 2H), 2.49–2.55 (m, 1H), 2.20-2.26 (m, 2H), 1.08–1.17 (m, 2H), 0.84–0.89 (m, 2H), 0.76–0.78 (m, 2H). ^13^C-NMR (DMSO-*d*_6_, 100 MHz) δ: 153.66, 144.97, 141.67, 136.81, 133.32, 129.10, 128.11, 127.78, 127.04, 125.22, 124.91, 122.51, 121.80, 115.60, 30.54, 23.40, 12.81, 7.26, 6.79.

*3-(3-Buten-1-yl)-4-(naphth-1-yl)-4H-1,2,4-triazole* (**5b**): Colorless oil; 14.02 g (75%). ^1^H-NMR (DMSO-*d*_6_, 400 MHz) δ: 8.73 (s, 1H), 8.16–8.20 (m, 1H), 8.11–8.13 (m, 1H), 7.60–7.71 (m, 4H), 7.15 (d, *J* = 8.0 Hz, 1H), 5.61–5.71 (m, 1H), 4.86–4.87 (m, 1H), 4.83 (d, *J* = 0.8 Hz, 1H), 2.54 (t, *J* = 7.6 Hz, 2H), 2.20–2.25 (m, 2H). ^13^C-NMR (DMSO-*d*_6_, 100 MHz) δ: 153.59, 144.89, 136.78, 133.66, 130.12, 129.85, 129.11, 128.49, 128.14, 127.13, 125.62, 125.60, 121.25, 115.62, 30.55, 23.44.

*3-(3-Buten-1-yl)-4-(4-methylnaphth-1-yl)-4H-1,2,4-triazole* (**5c**): Colorless oil; 15.01 g (76%). ^1^H-NMR (DMSO-*d*_6_, 400 MHz) δ: 8.69 (s, 1H), 8.18 (d, *J* = 8.4 Hz, 1H), 7.69 (t, *J* = 7.6 Hz, 1H), 7.62 (t, *J* = 7.6 Hz, 1H), 7.52–7.58 (m, 2H), 7.11 (d, *J* = 8.4 Hz, 1H), 5.63–5.70 (m, 1H), 4.83–4.87 (m, 2H), 2.75 (s, 3H), 2.52–2.53 (m, 2H), 2.20–2.25 (m, 2H). ^13^C-NMR (DMSO-*d*_6_, 100 MHz) δ: 153.68, 144.99, 136.93, 136.82, 132.52, 129.15, 128.17, 127.76, 127.02, 126.03, 125.19, 124.98, 121.75, 115.62, 30.57, 23.39, 19.01.

*3-(3-Buten-1-yl)-4-(4-ethylnaphth-1-yl)-4H-1,2,4-triazole* (**5d**): Colorless oil; 14.77 g (71%). ^1^H-NMR (DMSO-*d*_6_, 400 MHz) δ: 8.69 (s, 1H), 8.24 (d, *J* = 8.8 Hz, 1H), 7.66–7.70 (m, 1H), 7.58–7.63 (m, 2H), 7.54 (d, *J* = 7.2 Hz, 1H), 7.12 (d, *J* = 8.0 Hz, 1H), 5.62–5.72 (m, 1H), 4.86–4.88 (m, 1H), 4.83 (d, *J* = 1.2 Hz, 1H), 3.11–3.23 (m, 2H), 2.49–2.53 (m, 2H), 2.20–2.26 (m, 2H), 1.34 (t, *J* = 7.4 Hz, 3H). ^13^C-NMR (DMSO-*d*_6_, 100 MHz) δ: 153.69, 145.00, 142.66, 136.81, 131.74, 129.39, 128.18, 127.64, 127.04, 125.32, 124.50, 124.46, 121.94, 115.59, 30.55, 25.16, 23.42, 14.87.

*3-(3-Buten-1-yl)-4-(4-n-propylnaphth-1-yl)-4H-1,2,4-triazole* (**5e**): White solid; 16.61 g (76%); m.p. 92.5–94 °C. ^1^H-NMR (DMSO-*d*_6_, 400 MHz) δ: 8.70 (s, 1H), 8.24 (d, *J* = 8.4 Hz, 1H), 7.65–7.69 (m, 1H), 7.58–7.62 (m, 2H), 7.52 (d, *J* = 7.2 Hz, 1H), 7.11 (d, *J* = 8.0 Hz, 1H), 5.63–5.70 (m, 1H), 4.86–4.87 (m, 1H), 4.83 (d, *J* = 1.6 Hz, 1H), 3.06–3.18 (m, 2H), 2.49–2.53 (m, 2H), 2.19–2.25 (m, 2H), 1.71–1.77 (m, 2H), 1.00 (t, *J* = 7.2 Hz, 3H). ^13^C-NMR (DMSO-*d*_6_, 100 MHz) δ: 153.68, 145.02, 141.13, 136.84, 131.90, 129.44, 128.21, 127.63, 126.99, 125.51, 125.15, 124.70, 121.92, 115.63, 34.15, 30.56, 23.53, 23.43, 13.91.

*3-(3-Buten-1-yl)-4-(4-isopropylnaphth-1-yl)-4H-1,2,4-triazole* (**5f**): Colorless oil; 16.39 g (75%). ^1^H-NMR (DMSO-*d*_6_, 400 MHz) δ: 8.70 (s, 1H), 8.34 (d, *J* = 8.8 Hz, 1H), 7.56–7.72 (m, 4H), 7.12 (d, *J* = 8.0 Hz, 1H), 5.63–5.71 (m, 1H), 4.87–4.89 (m, 1H), 4.84 (d, *J* = 1.2 Hz, 1H), 3.82-3.88 (m, 1H), 2.52–2.54 (m, 2H), 2.22–2.27 (m, 2H), 1.41 (d, *J* = 6.8 Hz, 3H), 1.36 (d, *J* = 6.8 Hz, 3H). ^13^C-NMR (DMSO-*d*_6_, 100 MHz) δ: 154.39, 147.65, 145.74, 137.55, 132.05, 130.14, 128.75, 128.23, 127.75, 126.03, 124.75, 122.72, 122.22, 116.33, 31.24, 28.76, 24.30, 24.14, 23.66.

*3-(3-Buten-1-yl)-4-(4-methoxynaphth-1-yl)-4H-1,2,4-triazole* (**5g**): White solid; 15.29 g (73%); m.p. 92.5–94 °C. ^1^H-NMR (DMSO-*d*_6_, 400 MHz) δ: 8.66 (s, 1H), 8.27–8.30 (m, 1H), 7.60–7.65 (m, 3H), 7.11 (d, *J* = 8.4 Hz, 1H), 7.03–7.06 (m, 1H), 5.62–5.72 (m, 1H), 4.86–4.88 (m, 1H), 4.84 (d, *J* = 1.2 Hz, 1H), 4.05 (s, 3H), 2.48–2.53 (m, 2H), 2.20–2.26 (m, 2H). ^13^C-NMR (DMSO-*d*_6_, 100 MHz) δ: 155.93, 153.89, 145.19, 136.86, 130.00, 128.48, 126.44, 126.25, 125.02, 122.26, 122.22, 121.22, 115.60, 103.81, 56.06, 30.60, 23.37.

*3-(3-Buten-1-yl)-4-(4-ethoxynaphth-1-yl)-4H-1,2,4-triazole* (**5h**): White solid; 16.28 g (74%); m.p. 72.5–73.5 °C. ^1^H-NMR (DMSO-*d*_6_, 400 MHz) δ: 8.65 (s, 1H), 8.29–8.31 (m, 1H), 7.57–7.64 (m, 3H), 7.09 (d, *J* = 8 Hz, 1H), 7.02–7.05 (m, 1H), 5.61–5.72 (m, 1H), 4.86–4.88 (m, 1H), 4.83 (s, 1H), 4.30 (q, *J* = 6.9 Hz, 2H), 2.51–2.53 (m, 2H), 2.20–2.25 (m, 2H), 1.49 (t, *J* = 7.0 Hz, 3H). ^13^C-NMR (DMSO-*d*_6_, 100 MHz): 155.17, 153.88, 145.15, 136.82, 130.05, 128.39, 126.29, 126.24, 125.12, 122.30, 122.06, 121.16, 115.52, 104.36, 64.02, 30.58, 23.37, 14.42.

*4-(4-Bromonaphth-1-yl)-3-(3-buten-1-yl)-4H-1,2,4-triazole* (**5i**): White solid; 19.20 g (78%); m.p. 97.5–99 °C. ^1^H-NMR (DMSO-*d*_6_, 400 MHz) δ: 8.74 (s, 1H), 8.30 (d, *J* = 8.4 Hz, 1H), 8.09 (d, *J* = 8.0 Hz, 1H), 7.81–7.85 (m, 1H), 7.73 (t, *J* = 7.6 Hz, 1H), 7.65 (d, *J* = 7.6 Hz, 1H), 7.18 (d, *J* = 8.4 Hz, 1H), 5.62–5.72 (m, 1H), 4.86–4.88 (m, 1H), 4.84 (s, 1H), 2.52–2.56 (m, 2H), 2.21–2.27 (m, 2H). ^13^C-NMR (DMSO-*d*_6_, 100 MHz) δ: 153.61, 144.86, 136.75, 131.65, 130.34, 130.00, 129.75, 129.18, 129.03, 127.21, 126.39, 123.84, 122.29, 115.69, 30.52, 23.36.

*4-((4-Bromonaphth-1-yl)methyl)-3-(3-buten-1-yl)-4H-1,2,4-triazole* (**5j**): White solid; 19.76 g (77%); m.p. 120.5–122.5 °C. ^1^H-NMR (DMSO-*d*_6_, 400 MHz) δ: 8.44 (s, 1H), 8.22–8.24 (m, 1H), 8.13–8.16 (m, 1H), 7.86 (d, *J* = 7.6 Hz, 1H), 7.71–7.78 (m, 2H), 6.77 (d, *J* = 8.0 Hz, 1H), 5.74–5.85 (m, 3H), 4.90–4.99 (m, 2H), 2.72 (t, *J* = 7.6 Hz, 2H), 2.32–2.38 (m, 2H). ^13^C-NMR (DMSO-*d*_6_, 100 MHz) δ: 153.21, 144.43, 137.12, 132.41, 131.26, 131.12, 129.66, 128.06, 127.69, 127.17, 125.00, 123.81, 122.13, 115.52, 44.39, 30.45, 23.18.

*4-(2-(4-Bromonaphth-1-yl)ethyl)-3-(3-buten-1-yl)-4H-1,2,4-triazole* (**5k**): Colorless oil; 19.77 g (74%). ^1^H-NMR (DMSO-*d*_6_, 400 MHz) δ: 8.40 (s, 1H), 8.17-8.19 (m, 2H), 7.76 (d, *J* = 7.6 Hz, 1H), 7.65–7.73 (m, 2H), 7.12 (d, *J* = 7.6 Hz, 1H), 5.61–5.72 (m, 1H), 4.89 (s, 1H), 4.85–4.86 (m, 1H), 4.25 (t, *J* = 7.0 Hz, 2H), 3.49 (t, *J* = 7.0 Hz, 2H), 2.33–2.37 (m, 2H), 2.14–2.21 (m, 2H). ^13^C-NMR (DMSO-*d*_6_, 100 MHz) δ: 152.51, 143.66, 137.06, 134.32, 132.61, 131.23, 129.53, 127.75, 127.57, 127.30, 127.12, 124.28, 121.02, 115.27, 43.68, 32.89, 30.30, 22.71.

*4-((4-Bromonaphth-1-yl)methyl)-3-(4-hydroxybutyl)-4H-1,2,4-triazole* (**5l**): White solid; 19.72 g (73%); m.p. 126.5–128 °C. ^1^H-NMR (DMSO-*d*_6_, 400 MHz) δ: 8.43 (s, 1H), 8.21–8.24 (m, 1H), 8.13-8.15 (m, 1H), 7.85 (d, *J* = 7.6 Hz, 1H), 7.71–7.78 (m, 2H), 6.74 (d, *J* = 8.0 Hz, 1H), 5.74 (s, 2H), 4.35 (t, *J* = 5.0 Hz, 1H), 3.31–3.35 (m, 2H), 2.64 (t, *J* = 7.6 Hz, 2H), 1.58–1.66 (m, 2H), 1.38–1.45 (m, 2H). ^13^C-NMR (DMSO-*d*_6_, 100 MHz) δ: 153.86, 144.34, 132.49, 131.25, 131.13, 129.67, 128.06, 127.70, 127.19, 124.88, 123.78, 122.13, 60.23, 44.40, 31.84, 23.45, 23.17.

*4-((4-Bromonaphth-1-yl)methyl)-3-(5-hydroxypentyl)-4H-1,2,4-triazole* (**5m**): White solid; 20.77 g (74%); m.p. 88.5–91 °C. ^1^H-NMR (DMSO-*d*_6_, 400 MHz) δ: 8.43 (s, 1H), 8.22–8.24 (m, 1H), 8.14 (d, *J* = 8.8 Hz, 1H), 7.86 (d, *J* = 8.0 Hz, 1H), 7.71–7.78 (m, 2H), 6.75 (d, *J* = 8.0 Hz, 1H), 5.74 (s, 2H), 4.30 (t, *J* = 5.0 Hz, 1H), 3.29–3.33 (m, 2H), 2.62 (t, *J* = 7.4 Hz, 2H), 1.52–1.60 (m, 2H), 1.22–1.37 (m, 4H). ^13^C-NMR (DMSO-*d*_6_, 100 MHz) δ: 153.82, 144.35, 132.53, 131.26, 131.13, 129.69, 128.10, 127.72, 127.20, 124.92, 123.81, 122.10, 60.50, 44.39, 32.07, 26.45, 25.07, 23.65.

*4-((4-Bromonaphth-1-yl)methyl)-3-(hydroxymethyl)-4H-1,2,4-triazole* (**5n**): White solid; 18.85 g (79%); m.p. 197–200 °C. ^1^H-NMR (DMSO-*d*_6_, 400 MHz) δ: 8.40 (s, 1H), 8.22 (d, *J* = 8.4 Hz, 1H), 8.17 (d, *J* = 8.0 Hz, 1H), 7.86 (d, *J* = 7.6 Hz, 1H), 7.69–7.77 (m, 2H), 6.93 (d, *J* = 7.6 Hz, 1H), 5.82 (s, 2H), 5.75 (t, *J* = 5.6 Hz, 1H), 4.59 (d, *J* = 5.6 Hz, 2H). ^13^C-NMR (DMSO-*d*_6_, 100 MHz) δ: 154.07, 145.58, 132.97, 132.11, 131.84, 130.43, 128.75, 128.49, 127.89, 126.44, 124.53, 122.92, 54.32, 45.53.

*4-((4-Bromonaphth-1-yl)methyl)-3-((E)-1-propen-1-yl)-4H-1,2,4-triazole* (**5o**): White solid; 2.95 g (12%); m.p. 157–159 °C. ^1^H-NMR (DMSO-*d*_6_, 400 MHz) δ: 8.54 (s, 1H), 8.21–8.23 (m, 1H), 8.14–8.16 (m, 1H), 7.84 (d, *J* = 7.6 Hz, 1H), 7.71–7.78 (m, 2H), 6.67 (d, *J* = 7.6 Hz, 1H), 6.26 (dd, *J* = 1.6 Hz and 11.6 Hz, 1H), 6.06–6.14 (m, 1H), 5.80 (s, 2H), 2.11 (dd, *J* = 1.6 Hz and 7.2 Hz, 3H). ^13^C-NMR (DMSO-*d*_6_, 100 MHz) δ: 150.79, 143.82, 135.12, 132.61, 131.13, 131.06, 129.66, 128.07, 127.69, 127.15, 124.59, 123.72, 122.02, 112.61, 44.39, 15.69.

*3-Allyl-4-((4-bromonaphth-1-yl)methyl)-4H-1,2,4-triazole* (**5p**): White solid; 18.85 g (60%); m.p. 146.5–148 °C. ^1^H-NMR (DMSO-*d*_6_, 400 MHz) δ: 8.43 (s, 1H), 8.22–8.24 (m, 1H), 8.10–8.12 (m, 1H), 7.86 (d, *J* = 7.6 Hz, 1H), 7.71–7.78 (m, 2H), 6.79 (d, *J* = 7.6 Hz, 1H), 5.85–5.95 (m, 1H), 5.72 (s, 2H), 5.04 (s, 1H), 5.00–5.02 (m, 1H), 3.51 (d, *J* = 6.4 Hz, 2H). ^13^C-NMR (DMSO-*d*_6_, 100 MHz) δ: 151.87, 144.57, 132.61, 132.19, 131.27, 131.12, 129.68, 128.05, 127.72, 127.19, 125.20, 123.74, 122.17, 117.42, 44.54, 28.30.

*4-((4-Bromonaphth-1-yl)methyl)-3-(2-hydroxyethyl)-4H-1,2,4-triazole* (**5q**): White solid; 19.18 g (77%); m.p. 179–182 °C. ^1^H-NMR (DMSO-*d*_6_, 400 MHz) δ: 8.40 (s, 1H), 8.22–8.24 (m, 1H), 8.15 (d, *J* = 8.4 Hz, 1H), 7.85 (d, *J* = 7.6 Hz, 1H), 7.71–7.78 (m, 2H), 6.76 (d, *J* = 7.6 Hz, 1H), 5.78 (s, 2H), 4.83 (t, *J* = 5.4 Hz, 1H), 3.68–3.72 (m, 2H), 2.82 (t, *J* = 6.8 Hz, 2H). ^13^C-NMR (DMSO-*d*_6_, 100 MHz) δ: 152.36, 144.28, 132.45, 131.28, 131.14, 129.74, 128.12, 127.77, 127.20, 125.02, 123.83, 122.11, 59.11, 44.56, 27.65.

*4-((4-Bromonaphth-1-yl)methyl)-3-(2-hydroxypropyl)-4H-1,2,4-triazole* (**5r**): White solid; 18.70 g (72%); m.p. 155.5–157 °C. ^1^H-NMR (DMSO-*d*_6_, 400 MHz) δ: 8.36 (s, 1H), 8.22–8.24 (m, 1H), 8.13–8.15 (m, 1H), 7.85 (d, *J* = 8.0 Hz, 1H), 7.71–7.78 (m, 2H), 6.75 (d, *J* = 7.6 Hz, 1H), 5.80 (d, *J* = 17.6 Hz, 1H), 5.76 (d, *J* = 17.6 Hz, 1H), 4.86 (d, *J* = 4.4 Hz, 1H), 3.97–4.03 (m, 1H), 2.77 (dd, *J* = 5.6 Hz and 14.8 Hz, 1H), 2.70 (dd, *J* = 6.8 Hz and 14.8 Hz, 1H), 1.09 (d, *J* = 6.4 Hz, 3H). ^13^C-NMR (DMSO-*d*_6_, 100 MHz) δ: 152.34, 144.16, 132.47, 131.28, 131.12, 129.69, 128.06, 127.72, 127.17, 125.05, 123.83, 122.10, 65.16, 44.63, 33.52, 23.17.

*4-((4-Bromonaphth-1-yl)methyl)-4H-1,2,4-triazole* (**17**): White solid; 13.83 g (64%); m.p. 201–204 °C. ^1^H-NMR (DMSO-*d*_6_, 400 MHz) δ: 8.61 (s, 2H), 8.22 (d, *J* = 8.0 Hz, 2H), 7.90 (d, *J* = 7.6 Hz, 1H), 7.69–7.76 (m, 2H), 7.20 (d, *J* = 8.0 Hz, 1H), 5.79 (s, 2H). ^13^C-NMR (DMSO-*d*_6_, 100 MHz) δ: 143.42, 132.61, 131.47, 131.21, 129.77, 128.03, 127.83, 127.23, 126.87, 123.80, 122.57, 45.13.

#### 3.2.7. General Procedure for the Synthesis of **6a**–**6l**

To a stirred solution of **5a**–**5k** or **5o** (50 mmol) in THF/H_2_O (4/1 by *v*/*v*, 100 mL in total) at room temperature were added commercially available 50% solution of NMMO in water (23.43 g, 100 mmol) and a 0.16 M stock solution of OsO_4_ in *t*-BuOH/H_2_O (4/1 by *v*/*v*; 31.25 mL, 5 mmol). The resulting mixture was stirred at room temperature until the completion of reaction as indicated by TLC analysis (typically within 24 h). The reaction mixture was filtered off through celite via vacuum filtration and the filtrate was poured into ice-water (300 mL). The resulting mixture was extracted with CH_2_Cl_2_ (100 mL × 3). The combined extracts were washed successively with 1 M aqueous Na_2_S_2_O_3_ (100 mL) and 5% brine (100 mL), dried over anhydrous Na_2_SO_4_, and evaporated on a rotary evaporator to afford a residue, which was purified by column chromatography followed by trituration with EtOAc/*n*-hexane to produce the pure diol intermediates. These diol intermediates were not structurally characterized and were directly used in the next step.

The diol intermediates (deemed to be 50 mmol) were dissolved in THF/H_2_O (4/1 by *v*/*v*, 200 mL in total), followed by addition of NaIO_4_ (32.08 g, 150 mmol). The resulting mixture was stirred at room temperature until the completion of reaction as indicated by TLC analysis (typically within 2 h). The reaction mixture was poured into ice-water (400 mL) and the resulting mixture was extracted with CH_2_Cl_2_ (100 mL × 3). The combined extracts were washed with 5% brine (100 mL), dried over anhydrous Na_2_SO_4_, and evaporated on a rotary evaporator to afford a residue, which was purified by column chromatography to produce **6a**–**6l** after trituration with EtOAc/*n*-hexane if possible.

*3-(4-(4-Cyclopropylnaphth-1-yl)-4H-1,2,4-triazol-3-yl)propionaldehyde* (**6a**): White solid; 11.36 g (78%); m.p. 126.5–128.5 °C. ^1^H-NMR (DMSO-*d*_6_, 400 MHz) δ: 9.63 (s, 1H), 8.70 (s, 1H), 8.56 (d, *J* = 8.4 Hz, 1H), 7.71–7.75 (m, 1H), 7.62–7.66 (m, 1H), 7.58 (d, *J* = 8.0 Hz, 1H), 7.40 (d, *J* = 7.6 Hz, 1H), 7.15 (d, *J* = 8.4 Hz, 1H), 2.86 (t, *J* = 7.0 Hz, 2H), 2.63–2.71 (m, 1H), 2.51–2.59 (m, 2H), 1.11–1.14 (m, 2H), 0.84–0.90 (m, 1H), 0.79–0.80 (m, 1H). ^13^C-NMR (CDCl_3_, 100 MHz) δ: 199.79, 154.05, 144.70, 142.62, 134.17, 129.47, 127.92, 127.89, 127.12, 125.23, 124.74, 123.07, 121.80, 40.17, 17.15, 13.33, 6.80, 6.74.

*3-(4-(Naphth-1-yl)-4H-1,2,4-triazol-3-yl)propionaldehyde* (**6b**): White solid; 9.93 g (79%); m.p. 112–114 °C. ^1^H-NMR (DMSO-*d*_6_, 400 MHz) δ: 9.63 (s, 1H), 8.77 (s, 1H), 8.17–8.20 (m, 1H), 8.13 (d, *J* = 7.6 Hz, 1H), 7.61–7.71 (m, 4H), 7.18 (d, *J* = 8.4 Hz, 1H), 2.87 (t, *J* = 7.0 Hz, 2H), 2.66–2.73 (m, 1H), 2.49–2.61 (m, 1H). ^13^C-NMR (DMSO-*d*_6_, 100 MHz) δ: 201.58, 153.32, 145.03, 133.69, 130.17, 129.72, 129.05, 128.50, 128.20, 127.15, 125.64, 121.29, 39.38, 16.94.

*3-(4-(4-Methylnaphth-1-yl)-4H-1,2,4-triazol-3-yl)propionaldehyde* (**6c**): White solid; 10.08 g (76%); m.p. 101.5–103.5 °C. ^1^H-NMR (DMSO-*d*_6_, 400 MHz) δ: 9.62 (s, 1H), 8.71 (s, 1H), 8.18 (d, *J* = 8.4 Hz, 1H), 7.68–7.71 (m, 1H), 7.63 (t, *J* = 7.2 Hz, 1H), 7.53–7.59 (m, 2H), 7.15 (d, *J* = 8.0 Hz, 1H), 2.85 (t, *J* = 7.0 Hz, 2H), 2.75 (s, 3H), 2.64–2.73 (m, 1H), 2.52–2.59 (m, 1H). ^13^C-NMR (DMSO-*d*_6_, 100 MHz) δ: 201.59, 153.43, 145.14, 137.00, 132.57, 129.10, 128.06, 127.83, 127.04, 126.06, 125.23, 125.00, 121.79, 39.40, 19.02, 16.93.

*3-(4-(4-Ethylnaphth-1-yl)-4H-1,2,4-triazol-3-yl)propionaldehyde* (**6d**): White solid; 10.75 g (77%); m.p. 114–115.5 °C. ^1^H-NMR (DMSO-*d*_6_, 400 MHz) δ: 9.63 (s, 1H), 8.71 (s, 1H), 8.25 (d, *J* = 8.4 Hz, 1H), 7.69 (t, *J* = 7.6 Hz, 1H), 7.60–7.63 (m, 2H), 7.55 (d, *J* = 7.2 Hz, 1H), 7.16 (d, *J* = 8.4 Hz, 1H), 3.12–3.23 (m, 2H), 2.86 (t, *J* = 7.0 Hz, 2H), 2.64–2.72 (m, 1H), 2.52–2.59 (m, 1H), 1.35 (t, *J* = 7.6 Hz, 3H). ^13^C-NMR (DMSO-*d*_6_, 100 MHz) δ: 201.63, 153.43, 145.16, 142.74, 131.76, 129.33, 128.06, 127.72, 127.07, 125.36, 124.52, 121.97, 39.38, 25.18, 16.94, 14.93.

*3-(4-(4-n-Propylnaphth-1-yl)-4H-1,2,4-triazol-3-yl)propionaldehyde* (**6e**): White solid; 11.00 g (75%); m.p. 97.5–99.5 °C. ^1^H-NMR (DMSO-*d*_6_, 400 MHz) δ: 9.62 (s, 1H), 8.72 (s, 1H), 8.25 (d, *J* = 8.4 Hz, 1H), 7.66–7.70 (m, 1H), 7.59–7.63 (m, 2H), 7.53 (d, *J* = 7.6 Hz, 1H), 7.15 (d, *J* = 8.4 Hz, 1H), 3.05–3.20 (m, 2H), 2.85 (t, *J* = 7.0 Hz, 2H), 2.64–2.71 (m, 1H), 2.50–2.59 (m, 1H), 1.70-1.78 (m, 2H), 1.01 (t, *J* = 7.4 Hz, 3H). ^13^C-NMR (DMSO-*d*_6_, 100 MHz) δ: 201.62, 153.44, 145.16, 141.20, 131.95, 129.39, 128.09, 127.69, 127.01, 125.54, 125.19, 124.71, 121.95, 39.38, 34.18, 23.56, 16.94, 13.95.

*3-(4-(4-Isopropylnaphth-1-yl)-4H-1,2,4-triazol-3-yl)propionaldehyde* (**6f**): Colorless oil; 11.44 g (78%). ^1^H-NMR (DMSO-*d*_6_, 400 MHz) δ: 9.63 (s, 1H), 8.72 (s, 1H), 8.35 (d, *J* = 8.8 Hz, 1H), 7.60–7.71 (m, 4H), 7.16 (d, *J* = 8.0 Hz, 1H), 3.82–3.89 (m, 1H), 2.87 (t, *J* = 7.0 Hz, 2H), 2.64–2.72 (m, 1H), 2.53–2.59 (m, 1H), 1.41 (d, *J* = 6.8 Hz, 3H), 1.37 (d, *J* = 6.8 Hz, 3H). ^13^C-NMR (DMSO-*d*_6_, 100 MHz) δ: 201.57, 153.44, 147.00, 145.15, 131.39, 129.39, 127.94, 127.58, 127.05, 125.36, 124.05, 122.06, 121.57, 39.37, 28.08, 23.54, 22.99, 16.97.

*3-(4-(4-Methoxynaphth-1-yl)-4H-1,2,4-triazol-3-yl)propionaldehyde* (**6g**): White solid; 10.27 g (73%); m.p. 149–150.5 °C. ^1^H-NMR (DMSO-*d*_6_, 400 MHz) δ: 9.63 (s, 1H), 8.68 (s, 1H), 8.28–8.30 (m, 1H), 7.61–7.65 (m, 3H), 7.13 (d, *J* = 8.4 Hz, 1H), 7.07–7.10 (m, 1H), 4.06 (s, 3H), 2.85 (t, *J* = 7.0 Hz, 2H), 2.64–2.72 (m, 1H), 2.54–2.59 (m, 1H). ^13^C-NMR (DMSO-*d*_6_, 100 MHz) δ: 201.62, 155.98, 153.62, 145.33, 129.94, 128.53, 126.45, 126.28, 125.06, 122.23, 122.14, 121.25, 103.86, 56.08, 39.42, 16.90.

*3-(4-(4-Ethoxynaphth-1-yl)-4H-1,2,4-triazol-3-yl)propionaldehyde* (**6h**): White solid; 11.08 g (75%); m.p. 138.5–140 °C. ^1^H-NMR (DMSO-*d*_6_, 400 MHz) δ: 9.62 (s, 1H), 8.67 (s, 1H), 8.29–8.32 (m, 1H), 7.60–7.64 (m, 3H), 7.07–7.11 (m, 2H), 4.31 (q, *J* = 6.9 Hz, 2H), 2.84 (t, *J* = 7.0 Hz, 2H), 2.64–2.72 (m, 1H), 2.53–2.59 (m, 1H), 1.50 (t, *J* = 6.8 Hz, 3H). ^13^C-NMR (DMSO-*d*_6_, 100 MHz) δ: 201.61, 155.22, 153.62, 145.32, 129.99, 128.49, 126.35, 126.30, 125.16, 122.33, 121.94, 121.20, 104.47, 64.07, 39.42, 16.89, 14.46.

*3-(4-(4-Bromonaphth-1-yl)-4H-1,2,4-triazol-3-yl)propionaldehyde* (**6i**): White solid; 12.88 g (78%); m.p. 139–140.5 °C. ^1^H-NMR (DMSO-*d*_6_, 400 MHz) δ: 9.62 (s, 1H), 8.76 (s, 1H), 8.31 (d, *J* = 8.4 Hz, 1H), 8.10 (d, *J* = 8.0 Hz, 1H), 7.82–7.86 (m, 1H), 7.72–7.76 (m, 1H), 7.66 (d, *J* = 7.6 Hz, 1H), 7.23 (d, *J* = 8.4 Hz, 1H), 2.86 (t, *J* = 7.0 Hz, 2H), 2.67–2.74 (m, 1H), 2.56–2.62 (m, 1H). ^13^C-NMR (DMSO-*d*_6_, 100 MHz) δ: 201.54, 153.35, 144.97, 131.68, 130.30, 129.92, 129.77, 129.21, 129.02, 127.21, 126.41, 123.89, 122.34, 39.39, 16.91.

*3-(4-((4-Bromonaphth-1-yl)methyl)-4H-1,2,4-triazol-3-yl)propionaldehyde* (**6j**): White solid; 13.25 g (77%); m.p. 131.5–133 °C. ^1^H-NMR (DMSO-*d*_6_, 400 MHz) δ: 9.69 (s, 1H), 8.46 (s, 1H), 8.22–8.24 (m, 1H), 8.15–8.17 (m, 1H), 7.86 (d, *J* = 7.6 Hz, 1H), 7.72–7.79 (m, 2H), 6.78 (d, *J* = 7.6 Hz, 1H), 5.77 (s, 2H), 2.85–2.93 (m, 4H). ^13^C-NMR (DMSO-*d*_6_, 100 MHz) δ: 201.77, 152.91, 144.65, 132.19, 131.28, 131.15, 129.73, 128.11, 127.75, 127.20, 125.02, 123.83, 122.16, 44.41, 39.32, 16.88.

*3-(4-(2-(4-Bromonaphth-1-yl)ethyl)-4H-1,2,4-triazol-3-yl)propionaldehyde* (**6k**): White solid; 12.90 g (72%); m.p. 42.5–44 °C. ^1^H-NMR (DMSO-*d*_6_, 400 MHz) δ: 9.62 (s, 1H), 8.33 (s, 1H), 8.18–8.21 (m, 2H), 7.78 (d, *J* = 8.0 Hz, 1H), 7.65–7.73 (m, 2H), 7.18 (d, *J* = 7.6 Hz, 1H), 4.28 (t, *J* = 7.2 Hz, 2H), 3.50 (t, *J* = 7.4 Hz, 2H), 2.77 (t, *J* = 6.8 Hz, 2H), 2.67–2.71 (m, 2H). ^13^C-NMR (DMSO-*d*_6_, 100 MHz) δ: 201.74, 152.16, 143.94, 134.40, 132.64, 131.22, 129.60, 127.75, 127.66, 127.38, 127.11, 124.38, 120.97, 43.72, 39.39, 32.60, 16.46.

*(4-((4-Bromonaphth-1-yl)methyl)-4H-1,2,4-triazol-3-yl)carboxaldehyde* (**6l**): White solid; 12.01 g (76%); m.p. 211–213 °C. ^1^H-NMR (DMSO-*d*_6_, 400 MHz) δ: 10.06 (s, 1H), 8.84 (s, 1H), 8.22–8.25 (m, 1H), 8.13–8.15 (m, 1H), 7.83 (d, *J* = 7.6 Hz, 1H), 7.72–7.79 (m, 2H), 6.80 (d, *J* = 7.6 Hz, 1H), 6.04 (s, 2H). ^13^C-NMR (DMSO-*d*_6_, 100 MHz) δ: 182.52, 150.19, 147.54, 131.96, 131.20, 131.07, 129.69, 128.10, 127.90, 127.23, 125.09, 123.57, 122.29, 46.13.

#### 3.2.8. Synthesis of 1-(4-((4-Bromonaphth-1-yl)methyl)-4*H*-1,2,4-Triazol-3-yl)acetone **6n**

To a stirred solution of DMSO (2.34 g, 30 mmol) in dried CH_2_Cl_2_ (40 mL) cooled at −78 °C under N_2_ was added dropwise a solution of (COCl)_2_ (1.90 g, 15 mmol) in dried CH_2_Cl_2_ (10 mL) via syringe, and the resulting solution was stirred at this temperature for 0.5 h, followed by addition of a solution of **5r** (3.46 g, 10 mmol) in dried CH_2_Cl_2_ (10 mL) in a dropwise manner via syringe. After addition, the stirring was continued at this temperature for 1 h, and Et_3_N (6.07 g, 60 mmol) was added in a dropwise manner via syringe. The reaction mixture thus obtained was stirred at room temperature until the completion of reaction as indicated by TLC analysis (typically within 3 h).

The reaction mixture was poured into ice-water (200 mL). The resulting mixture was extracted with CH_2_Cl_2_ (100 mL × 3). The combined extracts were washed successively with saturated aqueous NaHCO_3_ (50 mL), 1 M hydrochloric acid (50 mL), and 5% brine (100 mL), dried over anhydrous Na_2_SO_4_, and evaporated on a rotary evaporator to afford a residue, which was purified by column chromatography to produce **6n**. White solid; 2.44 g (71%); m.p. 187–190 °C. ^1^H-NMR (DMSO-*d*_6_, 400 MHz) δ: 8.37 (s, 1H), 8.22 (d, *J* = 8.0 Hz, 1H), 8.08 (d, *J* = 7.6 Hz, 1H), 7.87 (d, *J* = 7.6 Hz, 1H), 7.69–7.77 (m, 2H), 6.90 (d, *J* = 7.6 Hz, 1H), 5.63 (s, 2H), 4.11 (s, 2H), 2.15 (s, 3H). ^13^C-NMR (DMSO-*d*_6_, 100 MHz) δ: 203.07, 148.91, 144.52, 131.92, 131.43, 131.16, 129.69, 128.05, 127.73, 127.17, 125.90, 123.92, 122.35, 44.89, 39.04, 29.47.

#### 3.2.9. General Procedure for the Synthesis of **7a**–**7k** and **17**

To a stirred solution of **6a**–**6l** (35 mmol) in *t*-BuOH (200 mL) and 2-methyl-2-butene (73.64 g, 1.05 mol) cooled with an ice-water bath was added a suspension of NaClO_2_ (80%; 11.87 g, 105 mmol) and NaH_2_PO_4_ (25.20 g, 210 mmol) in water (50 mL). The resulting mixture was stirred at room temperature until the completion of reaction as indicated by TLC analysis (typically within 6 h).

The reaction mixture was poured into ice-water (500 mL), and the mixture thus obtained was acidified (pH = 1–2) with concentrated hydrochloric acid and extracted with CH_2_Cl_2_ (100 mL × 3). The combined extracts were washed with water (100 mL), dried over anhydrous Na_2_SO_4_, and evaporated on a rotary evaporator to afford a residue, which was purified by column chromatography to produce **7a**–**7k** or **17** after trituration with EtOAc/*n*-hexane.

*3-(4-(4-Cyclopropylnaphth-1-yl)-4H-1,2,4-triazol-3-yl)propionic acid* (**7a**): White solid; 9.04 g (84%); m.p. 213.5–215 °C. ^1^H-NMR (DMSO-*d*_6_, 400 MHz) δ: 12.22 (brs, 1H), 8.71 (s, 1H), 8.56 (d, *J* = 8.8 Hz, 1H), 7.70–7.74 (m, 1H), 7.60–7.64 (m, 1H), 7.56 (d, *J* = 7.6 Hz, 1H), 7.41 (d, *J* = 7.6 Hz, 1H), 7.16 (d, *J* = 8.4 Hz, 1H), 2.58–2.67 (m, 3H), 2.45–2.55 (m, 2H), 1.08–1.17 (m, 2H), 0.73-0.91 (m, 2H). ^13^C-NMR (DMSO-*d*_6_, 100 MHz) δ: 173.01, 153.49, 145.04, 141.72, 133.37, 129.07, 128.05, 127.81, 127.07, 125.25, 124.92, 122.60, 121.93, 30.44, 19.51, 12.86, 7.18, 6.88.

*3-(4-(Naphth-1-yl)-4H-1,2,4-triazol-3-yl)propionic acid* (**7b**): White solid; 7.95 g (85%); m.p. 225–226.5 °C. ^1^H-NMR (DMSO-*d*_6_, 400 MHz) δ: 12.18 (brs, 1H), 8.74 (s, 1H), 8.18 (dd, *J* = 2.0 Hz and 7.2 Hz, 1H), 8.13 (d, *J* = 7.6 Hz, 1H), 7.59–7.72 (m, 4H), 7.19 (d, *J* = 8.0 Hz, 1H), 2.52–2.67 (m, 4H). ^13^C-NMR (DMSO-*d*_6_, 100 MHz) δ: 173.70, 154.11, 145.65, 134.39, 130.85, 130.47, 129.78, 129.18, 128.85, 127.85, 126.34, 122.09, 31.15, 20.22.

*3-(4-(4-Methylnaphth-1-yl)-4H-1,2,4-triazol-3-yl)propionic acid* (**7c**): White solid; 8.66 g (88%); m.p. 225 °C (dec). ^1^H-NMR (DMSO-*d*_6_, 400 MHz) δ: 12.17 (brs, 1H), 8.70 (s, 1H), 8.18 (d, *J* = 8.4 Hz, 1H), 7.69 (t, *J* = 7.6 Hz, 1H), 7.61 (t, *J* = 7.4 Hz, 1H), 7.53–7.57 (m, 2H), 7.15 (d, *J* = 8.4 Hz, 1H), 2.75 (s, 3H), 2.58–2.65 (m, 3H), 2.51–2.56 (m, 1H). ^13^C-NMR (DMSO-*d*_6_, 100 MHz) δ: 173.01, 153.54, 145.07, 137.02, 132.59, 129.13, 128.10, 127.81, 127.07, 126.08, 125.25, 125.01, 121.89, 30.48, 19.52, 19.05.

*3-(4-(4-Ethylnaphth-1-yl)-4H-1,2,4-triazol-3-yl)propionic acid* (**7d**): White solid; 8.48 g (82%); m.p. 206.5 °C (dec). ^1^H-NMR (DMSO-*d*_6_, 400 MHz) δ: 12.17 (brs, 1H), 8.70 (s, 1H), 8.25 (d, *J* = 8.4 Hz, 1H), 7.67–7.71 (m, 1H), 7.58–7.62 (m, 2H), 7.54 (d, *J* = 7.6 Hz, 1H), 7.16 (d, *J* = 8.0 Hz, 1H), 3.12–3.23 (m, 2H), 2.59–2.67 (m, 3H), 2.52–2.56 (m, 1H), 1.35 (t, *J* = 7.4 Hz, 3H). ^13^C-NMR (DMSO-*d*_6_, 100 MHz) δ: 173.70, 154.20, 145.76, 143.42, 132.46, 130.04, 128.79, 128.36, 127.76, 126.04, 125.20, 122.76, 31.12, 25.88, 20.20, 15.62.

*3-(4-(4-n-Propylnaphth-1-yl)-4H-1,2,4-triazol-3-yl)propionic acid* (**7e**): White solid; 9.10 g (84%); m.p. 171–173.5 °C. ^1^H-NMR (DMSO-*d*_6_, 400 MHz) δ: 12.15 (brs, 1H), 8.71 (s, 1H), 8.24 (d, *J* = 8.4 Hz, 1H), 7.68 (t, *J* = 7.6 Hz, 1H), 7.52–7.61 (m, 3H), 7.15 (d, *J* = 8.4 Hz, 1H), 3.04–3.19 (m, 2H), 2.60–2.64 (m, 2H), 2.49–2.56 (m, 2H), 1.74–1.76 (m, 2H), 1.01 (t, *J* = 7.2 Hz, 3H). ^13^C-NMR (DMSO-*d*_6_, 100 MHz) δ: 173.13, 153.53, 145.06, 141.18, 131.94, 129.40, 128.14, 127.64, 127.01, 125.53, 125.18, 124.69, 122.04, 34.19, 30.54, 23.57, 19.55, 13.97.

*3-(4-(4-Isopropylnaphth-1-yl)-4H-1,2,4-triazol-3-yl)propionic acid* (**7f**): White solid; 8.88 g (82%); m.p. 178–180.5 °C. ^1^H-NMR (DMSO-*d*_6_, 400 MHz) δ: 12.16 (brs, 1H), 8.71 (s, 1H), 8.34 (d, *J* = 8.8 Hz, 1H), 7.69 (t, *J* = 7.6 Hz, 1H), 7.58–7.64 (m, 3H), 7.16 (d, *J* = 8.4 Hz, 1H), 3.82–3.89 (m, 1H), 2.59–2.68 (m, 3H), 2.51–2.56 (m, 1H), 1.41 (d, *J* = 6.8 Hz, 3H), 1.37 (d, *J* = 6.8 Hz, 3H). ^13^C-NMR (DMSO-*d*_6_, 100 MHz) δ: 173.03, 153.52, 146.99, 145.08, 131.38, 129.40, 127.97, 127.54, 127.06, 125.36, 124.04, 122.15, 121.57, 30.41, 28.09, 23.57, 23.02, 19.53.

*3-(4-(4-Methoxynaphth-1-yl)-4H-1,2,4-triazol-3-yl)propionic acid* (**7g**): White solid; 8.74 g (84%); m.p. 243–245 °C. ^1^H-NMR (DMSO-*d*_6_, 400 MHz) δ: 12.15 (brs, 1H), 8.67 (s, 1H), 8.28–8.30 (m, 1H), 7.60–7.65 (m, 3H), 7.07–7.13 (m, 2H), 4.06 (s, 3H), 2.59–2.65 (m, 3H), 2.52–2.57 (m, 1H). ^13^C-NMR (DMSO-*d*_6_, 100 MHz) δ: 173.00, 155.98, 153.69, 145.24, 129.96, 128.49, 126.46, 126.28, 125.05, 122.22, 122.18, 121.33, 103.87, 56.09, 30.49, 19.47.

*3-(4-(4-Ethoxynaphth-1-yl)-4H-1,2,4-triazol-3-yl)propionic acid* (**7h**): White solid; 8.94 g (82%); m.p. 231–233 °C. ^1^H-NMR (DMSO-*d*_6_, 400 MHz) δ: 12.23 (brs, 1H), 8.66 (s, 1H), 8.29–8.32 (m, 1H), 7.57–7.64 (m, 3H), 7.07–7.11 (m, 2H), 4.30 (q, *J* = 6.9 Hz, 2H), 2.49–2.65 (m, 4H), 1.50 (t, *J* = 7.0 Hz, 3H). ^13^C-NMR (DMSO-*d*_6_, 100 MHz) δ: 173.10, 155.21, 153.72, 145.22, 130.01, 128.44, 126.35, 126.28, 125.14, 122.30, 121.99, 121.28, 104.47, 64.07, 30.59, 19.51, 14.47.

*3-(4-(4-Bromonaphth-1-yl)-4H-1,2,4-triazol-3-yl)propionic acid* (**7i**): White solid; 10.78 g (89%); m.p. 237–240 °C. ^1^H-NMR (DMSO-*d*_6_, 400 MHz) δ: 12.09 (brs, 1H), 8.75 (s, 1H), 8.30 (d, *J* = 8.4 Hz, 1H), 8.10 (d, *J* = 8.0 Hz, 1H), 7.83 (t, *J* = 7.6 Hz, 1H), 7.72 (t, *J* = 7.6 Hz, 1H), 7.64 (d, *J* = 8.0 Hz, 1H), 7.23 (d, *J* = 8.4 Hz, 1H), 2.52-2.69 (m, 4H). ^13^C-NMR (DMSO-*d*_6_, 100 MHz) δ: 173.01, 153.46, 144.89, 131.68, 130.32, 129.97, 129.78, 129.17, 129.04, 127.21, 126.41, 123.87, 122.44, 30.49, 19.48.

*3-(4-((4-Bromonaphth-1-yl)methyl)-4H-1,2,4-triazol-3-yl)propionic acid* (**7j**): White solid; 10.59 g (84%); m.p. 225–227 °C. ^1^H-NMR (DMSO-*d*_6_, 400 MHz) δ: 12.18 (brs, 1H), 8.43 (s, 1H), 8.23 (d, *J* = 7.6 Hz, 1H), 8.14–8.16 (m, 1H), 7.85 (d, *J* = 7.6 Hz, 1H), 7.72–7.75 (m, 2H), 6.78 (d, *J* = 7.6 Hz, 1H), 5.76 (s, 2H), 2.81–2.85 (m, 2H), 2.68–2.72 (m, 2H). ^13^C-NMR (DMSO-*d*_6_, 100 MHz) δ: 173.22, 152.99, 144.50, 132.23, 131.29, 131.14, 129.72, 128.12, 127.76, 127.20, 125.04, 123.85, 122.12, 44.36, 30.45, 19.35.

*3-(4-(2-(4-Bromonaphth-1-yl)ethyl)-4H-1,2,4-triazol-3-yl)propionic acid* (**7k**): White solid; 10.61 g (81%); m.p. 210–213 °C. ^1^H-NMR (DMSO-*d*_6_, 400 MHz) δ: 12.18 (brs, 1H), 8.31 (s, 1H), 8.18–8.21 (m, 2H), 7.78 (d, *J* = 7.6 Hz, 1H), 7.65–7.73 (m, 2H), 7.18 (d, *J* = 7.6 Hz, 1H), 4.28 (t, *J* = 7.2 Hz, 2H), 3.49 (t, *J* = 7.2 Hz, 2H), 2.69 (t, *J* = 6.6 Hz, 2H), 2.61 (t, *J* = 6.4 Hz, 2H). ^13^C-NMR (DMSO-*d*_6_, 100 MHz) δ: 173.25, 152.34, 143.82, 134.41, 132.66, 131.24, 129.60, 127.74, 127.65, 127.38, 127.13, 124.39, 120.99, 43.72, 32.59, 30.64, 18.96.

*4-((4-Bromonaphth-1-yl)methyl)-4H-1,2,4-triazole* (**17**): 4.13 g (41%). The physical properties of the sample **17** obtained by this procedure were in good agreement with those for the sample obtained from **3i**–**3k** described above.

#### 3.2.10. General Procedure for the Synthesis of **7l**, **7m** and **17**

To a stirred solution of **5l**–**5n** (35 mmol) and (2,2,6,6-tetramethylpiperidin-1-yl)oxyl (TEMPO; 0.55 g, 3.5 mmol) in MeCN (200 mL) and phosphate buffer (150 mL, pH = 6.7; prepared by mixing aqueous 0.67 M Na_2_HPO_4_ and 0.67 M NaH_2_PO_4_ in a ratio of 1/1) at 35 °C were added aqueous NaClO_2_ and aqueous NaClO simultaneously over 2 h. The aqueous NaClO_2_ solution was prepared by dissolving 80% solid NaClO_2_ (80%; 7.91 g, 70 mmol) in water (40 mL), while the aqueous NaClO solution was prepared by diluting a commercially available aqueous NaClO solution (21% by *w*/*w*; 0.35 mL, 1 mmol) with water (20 mL). After addition, the reaction mixture was stirred at 35 °C until the completion of reaction as indicated by TLC analysis (typically within 10 h).

The reaction mixture was poured into ice-water (200 mL), and the aqueous mixture thus obtained was acidified (pH = 1–2) with concentrated hydrochloric acid and extracted with CH_2_Cl_2_ (100 mL × 3). The combined extracts were washed successively with 1% aqueous Na_2_S_2_O_3_ (100 mL) and water (100 mL), dried over anhydrous Na_2_SO_4_, and evaporated on a rotary evaporator to afford a residue, which was purified by column chromatography through a short silica gel column to produce **7l**, **7m** or **17** after trituration with EtOAc/*n*-hexane.

*4-(4-((4-Bromonaphth-1-yl)methyl)-4H-1,2,4-triazol-3-yl)butanoic acid* (**7l**): White solid; 10.74 g (82%); m.p. 175–177.5 °C. ^1^H-NMR (DMSO-*d*_6_, 400 MHz) δ: 12.03 (brs, 1H), 8.44 (s, 1H), 8.22–8.24 (m, 1H), 8.13–8.15 (m, 1H), 7.85 (d, *J* = 7.6 Hz, 1H), 7.72–7.78 (m, 2H), 6.72 (d, *J* = 7.6 Hz, 1H), 5.74 (s, 2H), 2.68 (t, *J* = 7.6 Hz, 2H), 2.29 (t, *J* = 7.2 Hz, 2H), 1.81–1.89 (m, 2H). ^13^C-NMR (DMSO-*d*_6_, 100 MHz) δ: 174.02, 153.43, 144.45, 132.46, 131.24, 131.14, 129.74, 128.11, 127.74, 127.21, 124.74, 123.80, 122.11, 44.41, 32.72, 22.93, 21.93.

*5-(4-((4-Bromonaphth-1-yl)methyl)-4H-1,2,4-triazol-3-yl)pentanoic acid* (**7m**): White solid; 11.01 g (81%); m.p. 179.5–181 °C. ^1^H-NMR (DMSO-*d*_6_, 400 MHz) δ: 11.99 (brs, 1H), 8.42 (s, 1H), 8.21–8.24 (m, 1H), 8.13–8.15 (m, 1H), 7.85 (d, *J* = 8.0 Hz, 1H), 7.71–7.78 (m, 2H), 6.76 (d, *J* = 7.6 Hz, 1H), 5.74 (s, 2H), 2.65 (t, *J* = 7.4 Hz, 2H), 2.16 (t, *J* = 7.2 Hz, 2H), 1.58–1.65 (m, 2H), 1.47–1.54 (m, 2H). ^13^C-NMR (DMSO-*d*_6_, 100 MHz) δ: 174.29, 153.65, 144.38, 132.46, 131.30, 131.17, 129.72, 128.10, 127.74, 127.23, 124.99, 123.82, 122.18, 44.44, 33.28, 25.96, 24.01, 23.36.

*4-((4-Bromonaphth-1-yl)methyl)-4H-1,2,4-triazole* (**17**): 3.43 g (34%). The physical properties of sample **17** obtained by this procedure were in good agreement with those for the sample obtained from **3i**–**3k** described above.

#### 3.2.11. General Procedure for the Synthesis of **8a**–**8m**

To a stirred mixture of **7a**–**7m** (28 mmol) in dried CH_2_Cl_2_ (100 mL) cooled in an ice-water bath were added EDCI (8.05 g, 42 mmol), DMAP (1.71 g, 14 mmol) and MeOH (8.97 g, 280 mmol), and the resulting mixture was stirred at room temperature under N_2_ until the completion of reaction as indicated by TLC analysis (typically within 6 h).

The reaction mixture was diluted with CH_2_Cl_2_ (200 mL) and washed successively with 1 M hydrochloric acid (100 mL), saturated aqueous Na_2_CO_3_ (100 mL), and 5% brine (100 mL). The organic phase was dried over anhydrous Na_2_SO_4_ and evaporated on a rotary evaporator to afford a residue, which was purified by column chromatography to produce **8a**–**8m** after trituration with EtOAc/*n*-hexane if possible. 

*Methyl 3-(4-(4-cyclopropylnaphth-1-yl)-4H-1,2,4-triazol-3-yl)propionate* (**8a**): White solid; 6.93 g (77%); m.p. 88.5–89.5 °C. ^1^H-NMR (DMSO-*d*_6_, 400 MHz) δ: 8.70 (s, 1H), 8.56 (d, *J* = 8.4 Hz, 1H), 7.72 (t, *J* = 7.6 Hz, 1H), 7.64 (t, *J* = 7.4 Hz, 1H), 7.56 (d, *J* = 7.6 Hz, 1H), 7.41 (d, *J* = 7.6 Hz, 1H), 7.15 (d, *J* = 8.4 Hz, 1H), 3.51 (s, 3H), 2.63–2.75 (m, 3H), 2.51–2.59 (m, 2H), 1.09–1.16 (m, 2H), 0.85–0.88 (m, 1H), 0.76–0.79 (m, 1H). ^13^C-NMR (DMSO-*d*_6_, 100 MHz) δ: 171.99, 153.26, 145.09, 141.76, 133.36, 129.05, 127.97, 127.84, 127.09, 125.27, 124.94, 122.59, 121.87, 51.37, 30.11, 19.39, 12.85, 7.20, 6.89.

*Methyl 3-(4-(naphth-1-yl)-4H-1,2,4-triazol-3-yl)propionate* (**8b**): White solid; 5.99 g (76%); m.p. 129–130.5 °C. ^1^H-NMR (DMSO-*d*_6_, 400 MHz) δ: 8.75 (s, 1H), 8.17–8.20 (m, 1H), 8.13 (d, *J* = 7.6 Hz, 1H), 7.61–7.72 (m, 4H), 7.18 (d, *J* = 8.0 Hz, 1H), 3.51 (s, 3H), 2.65–2.75 (m, 3H), 2.54–2.63 (m, 1H). ^13^C-NMR (DMSO-*d*_6_, 100 MHz) δ: 171.97, 153.16, 145.00, 133.69, 130.18, 129.69, 129.05, 128.49, 128.18, 127.16, 125.65, 125.63, 121.32, 51.36, 30.12, 19.40.

*Methyl 3-(4-(4-methylnaphth-1-yl)-4H-1,2,4-triazol-3-yl)propionate* (**8c**): White solid; 6.37 g (77%); m.p. 138.5–140.5 °C. ^1^H-NMR (DMSO-*d*_6_, 400 MHz) δ: 8.70 (s, 1H), 8.19 (d, *J* = 8.0 Hz, 1H), 7.70 (dt, *J* = 1.2 Hz and 8.4 Hz, 1H), 7.63 (dt, *J* = 1.2 Hz and 6.8 Hz, 1H), 7.53–7.58 (m, 2H), 7.15 (d, *J* = 8.0 Hz, 1H), 3.51 (s, 3H), 2.75 (s, 3H), 2.70–2.74 (m, 2H), 2.63–2.69 (m, 1H), 2.52–2.59 (m, 1H). ^13^C-NMR (DMSO-*d*_6_, 100 MHz) δ: 171.96, 153.26, 145.10, 137.01, 132.55, 129.09, 128.02, 127.80, 127.04, 126.05, 125.22, 124.99, 121.81, 51.36, 30.13, 19.38, 19.02.

*Methyl 3-(4-(4-ethylnaphth-1-yl)-4H-1,2,4-triazol-3-yl)propionate* (**8d**): White solid; 6.84 g (79%); m.p. 116.5–118 °C. ^1^H-NMR (DMSO-*d*_6_, 400 MHz) δ: 8.71 (s, 1H), 8.25 (d, *J* = 8.4 Hz, 1H), 7.67–7.71 (m, 1H), 7.59–7.63 (m, 2H), 7.55 (d, *J* = 7.2 Hz, 1H), 7.15 (d, *J* = 8.0 Hz, 1H), 3.51 (s, 3H), 3.14–3.21 (m, 2H), 2.70–2.75 (m, 2H), 2.64–2.67 (m, 1H), 2.54–2.59 (m, 1H), 1.35 (t, *J* = 7.6 Hz, 3H). ^13^C-NMR (DMSO-*d*_6_, 100 MHz) δ: 171.97, 153.26, 145.10, 142.74, 131.75, 129.32, 128.03, 127.68, 127.06, 125.34, 124.51, 122.00, 51.35, 30.11, 25.17, 19.39, 14.91.

*Methyl 3-(4-(4-n-propylnaphth-1-yl)-4H-1,2,4-triazol-3-yl)propionate* (**8e**): White solid; 6.88 g (76%); m.p. 116.5–118 °C. ^1^H-NMR (DMSO-*d*_6_, 400 MHz) δ: 8.72 (s, 1H), 8.25 (d, *J* = 8.4 Hz, 1H), 7.68 (t, *J* = 7.6 Hz, 1H), 7.58–7.63 (m, 2H), 7.53 (d, *J* = 7.6 Hz, 1H), 7.15 (d, *J* = 8.0 Hz, 1H), 3.51 (s, 3H), 3.06–3.17 (m, 2H), 2.69–2.74 (m, 2H), 2.63–2.67 (m, 1H), 2.54–2.59 (m, 1H), 1.71–1.78 (m, 2H), 1.01 (t, *J* = 7.4 Hz, 3H). ^13^C-NMR (DMSO-*d*_6_, 100 MHz) δ: 171.96, 153.25, 145.09, 141.19, 131.93, 129.37, 128.06, 127.64, 126.99, 125.52, 125.16, 124.68, 121.97, 51.34, 34.18, 30.10, 23.55, 19.39, 13.93.

*Methyl 3-(4-(4-isopropylnaphth-1-yl)-4H-1,2,4-triazol-3-yl)propionate* (**8f**): White solid; 6.88 g (76%); m.p. 80–81.5 °C. ^1^H-NMR (DMSO-*d*_6_, 400 MHz) δ: 8.71 (s, 1H), 8.34 (d, *J* = 8.4 Hz, 1H), 7.69 (t, *J* = 7.6 Hz, 1H), 7.60–7.64 (m, 3H), 7.15 (d, *J* = 8.4 Hz, 1H), 3.82–3.88 (m, 1H), 3.51 (s, 3H), 2.71–2.76 (m, 2H), 2.64–2.70 (m, 1H), 2.53–2.59 (m, 1H), 1.41 (d, *J* = 6.8 Hz, 3H), 1.37 (d, *J* = 6.4 Hz, 3H). ^13^C-NMR (DMSO-*d*_6_, 100 MHz) δ: 171.97, 153.26, 146.99, 145.10, 131.36, 129.36, 127.89, 127.54, 127.04, 125.34, 124.03, 122.07, 121.55, 51.33, 30.07, 28.05, 23.54, 22.99, 19.39.

*Methyl 3-(4-(4-methoxynaphth-1-yl)-4H-1,2,4-triazol-3-yl)propionate* (**8g**): White solid; 6.80 g (78%); m.p. 134.5–135.5 °C. ^1^H-NMR (DMSO-*d*_6_, 400 MHz) δ: 8.67 (s, 1H), 8.28–8.30 (m, 1H), 7.60–7.65 (m, 3H), 7.13 (d, *J* = 8.4 Hz, 1H), 7.06–7.10 (m, 1H), 4.06 (s, 3H), 3.51 (s, 3H), 2.52–2.74 (m, 4H). ^13^C-NMR (DMSO-*d*_6_, 100 MHz) δ: 171.99, 156.00, 153.46, 145.29, 129.95, 128.51, 126.45, 126.28, 125.05, 122.23, 122.11, 121.28, 103.86, 56.08, 51.36, 30.15, 19.36.

*Methyl 3-(4-(4-ethoxynaphth-1-yl)-4H-1,2,4-triazol-3-yl)propionate* (**8h**): White solid; 6.92 g (76%); m.p. 117–118.5 °C. ^1^H-NMR (DMSO-*d*_6_, 400 MHz) δ: 8.67 (s, 1H), 8.29–8.32 (m, 1H), 7.61–7.65 (m, 2H), 7.59 (d, *J* = 8.0 Hz, 1H), 7.11 (d, *J* = 8.0 Hz, 1H), 7.06–7.09 (m, 1H), 4.31 (q, *J* = 6.9 Hz, 2H), 3.51 (s, 3H), 2.64–2.74 (m, 3H), 2.52–2.61 (m, 1H), 1.50 (t, *J* = 7.0 Hz, 3H). ^13^C-NMR (DMSO-*d*_6_, 100 MHz) δ: 171.96, 155.22, 153.43, 145.27, 129.98, 128.45, 126.34, 126.27, 125.13, 122.30, 121.90, 121.21, 104.45, 64.06, 51.33, 30.14, 19.34, 14.45.

*Methyl 3-(4-(4-bromonaphth-1-yl)-4H-1,2,4-triazol-3-yl)propionate* (**8i**): White solid; 8.07 g (80%); m.p. 126–127.5 °C. ^1^H-NMR (DMSO-*d*_6_, 400 MHz) δ: 8.76 (s, 1H), 8.31 (d, *J* = 8.4 Hz, 1H), 8.10 (d, *J* = 7.6 Hz, 1H), 7.82–7.86 (m, 1H), 7.72–7.76 (m, 1H), 7.65 (d, *J* = 8.0 Hz, 1H), 7.22 (d, *J* = 8.4 Hz, 1H), 3.51 (s, 3H), 2.68–2.75 (m, 3H), 2.53–2.63 (m, 1H). ^13^C-NMR (DMSO-*d*_6_, 100 MHz) δ: 171.94, 153.19, 144.93, 131.67, 130.29, 129.88, 129.77, 129.18, 129.02, 127.20, 126.39, 123.89, 122.35, 51.36, 30.12, 19.35.

*Methyl 3-(4-((4-bromonaphth-1-yl)methyl)-4H-1,2,4-triazol-3-yl)propionate* (**8j**): White solid; 8.07 g (77%); m.p. 100–101.5 °C. ^1^H-NMR (DMSO-*d*_6_, 400 MHz) δ: 8.45 (s, 1H), 8.22–8.25 (m, 1H), 8.14–8.17 (m, 1H), 7.86 (d, *J* = 8.0 Hz, 1H), 7.72–7.79 (m, 2H), 6.77 (d, *J* = 8.0 Hz, 1H), 5.77 (s, 2H), 3.56 (s, 3H), 2.85–2.88 (m, 2H), 2.75–2.79 (m, 2H). ^13^C-NMR (DMSO-*d*_6_, 100 MHz) δ: 172.20, 152.74, 144.60, 132.22, 131.26, 131.13, 129.71, 128.12, 127.75, 127.19, 124.96, 123.84, 122.12, 51.37, 44.35, 30.07, 19.26.

*Methyl 3-(4-(2-(4-bromonaphth-1-yl)ethyl)-4H-1,2,4-triazol-3-yl)propionate* (**8k**): White solid; 8.15 g (75%); m.p. 112.5–114 °C. ^1^H-NMR (DMSO-*d*_6_, 400 MHz) δ: 8.33 (s, 1H), 8.18–8.20 (m, 2H), 7.78 (d, *J* = 7.6 Hz, 1H), 7.65–7.73 (m, 2H), 7.16 (d, *J* = 7.6 Hz, 1H), 4.28 (t, *J* = 7.2 Hz, 2H), 3.56 (s, 3H), 3.49 (t, *J* = 7.2 Hz, 2H), 2.60–2.68 (m, 4H). ^13^C-NMR (DMSO-*d*_6_, 100 MHz) δ: 172.17, 152.03, 143.88, 134.38, 132.62, 131.23, 129.58, 127.74, 127.63, 127.36, 127.12, 124.35, 121.00, 51.37, 43.70, 32.64, 30.24, 18.83.

*Methyl 4-(4-((4-bromonaphth-1-yl)methyl)-4H-1,2,4-triazol-3-yl)butanoate* (**8l**): white foam; 8.59 g (79%). ^1^H-NMR (DMSO-*d*_6_, 400 MHz) δ: 8.44 (s, 1H), 8.22–8.24 (m, 1H), 8.13–8.15 (m, 1H), 7.85 (d, *J* = 7.6 Hz, 1H), 7.72–7.79 (m, 2H), 6.71 (d, *J* = 7.6 Hz, 1H), 5.73 (s, 2H), 3.51 (s, 3H), 2.67 (t, *J* = 7.4 Hz, 2H), 2.37 (t, *J* = 7.2 Hz, 2H), 1.83–1.90 (m, 2H). ^13^C-NMR (DMSO-*d*_6_, 100 MHz) δ: 172.88, 153.27, 144.46, 132.48, 131.23, 131.13, 129.74, 128.15, 127.76, 127.20, 124.76, 123.82, 122.07, 51.18, 44.37, 32.36, 22.82, 21.81.

*Methyl 5-(4-((4-bromonaphth-1-yl)methyl)-4H-1,2,4-triazol-3-yl)pentanoate* (**8m**): Colorless oil; 9.01 g (80%). ^1^H-NMR (DMSO-*d*_6_, 400 MHz) δ: 8.43 (s, 1H), 8.22–8.24 (m, 1H), 8.13–8.15 (m, 1H), 7.85 (d, *J* = 7.6 Hz, 1H), 7.72–7.78 (m, 2H), 6.74 (d, *J* = 7.6 Hz, 1H), 5.73 (s, 2H), 3.53 (s, 3H), 2.64 (t, *J* = 7.2 Hz, 2H), 2.24 (t, *J* = 7.2 Hz, 2H), 1.50–1.61 (m, 4H). ^13^C-NMR (DMSO-*d*_6_, 100 MHz) δ: 173.09, 153.54, 144.38, 132.52, 131.26, 131.13, 129.71, 128.13, 127.73, 127.20, 124.90, 123.83, 122.08, 51.11, 44.36, 32.81, 25.83, 23.86, 23.22.

#### 3.2.12. General Procedure for the Synthesis of **9a**–**9c** and **9f**–**9q**

A mixture of **8a**–**8m** (6 mmol) and *N*-halosuccinimide (NCS, NBS or NIS; 7.2 mmol) in MeCN (30 mL) was stirred at room temperature (**9b**, **9d**–**9l** or **9n**–**9q**), 60 °C (for **9m**), or reflux (**9a** and **9c**), until the completion of reaction as indicated by TLC analysis (typically within 24 h).

The reaction mixture was poured into ice-water (100 mL) and the aqueous mixture thus obtained was extracted with CH_2_Cl_2_ (50 mL × 3). The combined extracts were washed successively with 5% aqueous Na_2_S_2_O_3_ (50 mL), saturated aqueous Na_2_CO_3_ (50 mL × 3), and 5% brine (50 mL), dried over anhydrous Na_2_SO_4_, and evaporated on a rotary evaporator to afford a residue, which was purified by column chromatography to produce **9a**–**9c** or **9f**–**9q** after trituration with EtOAc/*n*-hexane if possible.

*Methyl 3-(5-chloro-4-(4-cyclopropylnaphth-1-yl)-4H-1,2,4-triazol-3-yl)propionate* (**9a**): White solid; 1.26 g (59%); m.p. 104–105.5 °C. ^1^H-NMR (DMSO-*d*_6_, 400 MHz) δ: 8.58 (d, *J* = 8.4 Hz, 1H), 7.74 (t, *J* = 7.6 Hz, 1H), 7.64–7.68 (m, 2H), 7.44 (d, *J* = 7.6 Hz, 1H), 7.15 (d, *J* = 8.0 Hz, 1H), 3.52 (s, 3H), 2.62–2.73 (m, 3H), 2.51–2.57 (m, 2H), 1.12–1.16 (m, 2H), 0.84–0.87 (m, 2H). ^13^C-NMR (DMSO-*d*_6_, 100 MHz) δ: 171.76, 155.96, 142.81, 141.45, 133.44, 128.71, 128.17, 127.21, 126.48, 126.14, 125.13, 122.67, 121.42, 51.39, 29.38, 20.45, 12.83, 7.23, 7.14.

*Methyl 3-(5-bromo-4-(4-cyclopropylnaphth-1-yl)-4H-1,2,4-triazol-3-yl)propionate* (**9b**): White solid; 1.61 g (67%); m.p. 122.5–123.5 °C. ^1^H-NMR (DMSO-*d*_6_, 400 MHz) δ: 8.58 (d, *J* = 8.4 Hz, 1H), 7.73 (t, *J* = 7.6 Hz, 1H), 7.62–7.67 (m, 2H), 7.44 (d, *J* = 7.6 Hz, 1H), 7.10 (d, *J* = 8.4 Hz, 1H), 3.51 (s, 3H), 2.62–2.72 (m, 3H), 2.51–2.56 (m, 2H), 1.12–1.16 (m, 2H), 0.84–0.87 (m, 2H). ^13^C-NMR (DMSO-*d*_6_, 100 MHz) δ: 171.78, 156.41, 142.66, 133.42, 130.48, 128.82, 128.09, 127.18, 127.01, 126.53, 125.11, 122.68, 121.58, 51.40, 29.51, 20.42, 12.84, 7.23, 7.16.

*Methyl 3-(4-(4-cyclopropylnaphth-1-yl)-5-iodo-4H-1,2,4-triazol-3-yl)propionate* (**9c**): White solid; 1.37 g (51%); m.p. 138.5–140 °C. ^1^H-NMR (DMSO-*d*_6_, 400 MHz) δ: 8.58 (d, *J* = 8.4 Hz, 1H), 7.73 (t, *J* = 7.6 Hz, 1H), 7.64 (t, *J* = 7.2 Hz, 1H), 7.55 (d, *J* = 7.6 Hz, 1H), 7.43 (d, *J* = 7.6 Hz, 1H), 7.02 (d, *J* = 8.4 Hz, 1H), 3.51 (s, 3H), 2.62–2.71 (m, 3H), 2.52–2.57 (m, 2H), 1.12–1.17 (m, 2H), 0.85–0.88 (m, 2H). ^13^C-NMR (DMSO-*d*_6_, 100 MHz) δ: 171.83, 156.29, 142.38, 133.41, 129.07, 128.42, 127.91, 127.12, 126.62, 125.06, 122.65, 121.89, 106.22, 51.38, 29.82, 20.33, 12.85, 7.26, 7.18.

*Methyl 3-(5-bromo-4-(naphth-1-yl)-4H-1,2,4-triazol-3-yl)propionate* (**9f**): White solid; 1.34 g (62%); m.p. 152–154 °C. ^1^H-NMR (DMSO-*d*_6_, 400 MHz) δ: 8.22–8.25 (m, 1H), 8.14–8.16 (m, 1H), 7.72–7.76 (m, 2H), 7.63–7.70 (m, 2H), 7.13 (d, *J* = 8.0 Hz, 1H), 3.52 (s, 3H), 2.65–2.73 (m, 3H), 2.51–2.58 (m, 1H). ^13^C-NMR (DMSO-*d*_6_, 100 MHz) δ: 171.78, 156.34, 133.77, 131.01, 130.29, 128.89, 128.78, 128.68, 128.48, 127.30, 126.92, 125.82, 121.04, 51.42, 29.53, 20.43.

*Methyl 3-(5-bromo-4-(4-methylnaphth-1-yl)-4H-1,2,4-triazol-3-yl)propionate* (**9g**): White solid; 1.37 g (61%); m.p. 127–128.5 °C. ^1^H-NMR (DMSO-*d*_6_, 400 MHz) δ: 8.20 (d, *J* = 8.4 Hz, 1H), 7.68–7.72 (m, 1H), 7.57–7.66 (m, 3H), 7.10 (d, *J* = 8.4 Hz, 1H), 3.51 (s, 3H), 2.76 (s, 3H), 2.63–2.72 (m, 3H), 2.51–2.56 (m, 1H). ^13^C-NMR (DMSO-*d*_6_, 100 MHz) δ: 171.78, 156.43, 138.01, 132.68, 130.52, 128.89, 128.09, 127.17, 127.12, 126.49, 126.28, 125.21, 121.54, 51.42, 29.56, 20.44, 19.08.

*Methyl 3-(5-bromo-4-(4-ethylnaphth-1-yl)-4H-1,2,4-triazol-3-yl)propionate* (**9h**): White solid; 1.47 g (63%); m.p. 107.5–109 °C. ^1^H-NMR (DMSO-*d*_6_, 400 MHz) δ: 8.27 (d, *J* = 8.4 Hz, 1H), 7.58–7.72 (m, 4H), 7.10 (d, *J* = 8.4 Hz, 1H), 3.52 (s, 3H), 3.19 (q, *J* = 7.6 Hz, 2H), 2.63–2.72 (m, 3H), 2.52–2.57 (m, 1H), 1.37 (t, *J* = 7.4 Hz, 3H). ^13^C-NMR (DMSO-*d*_6_, 100 MHz) δ: 171.80, 156.44, 143.57, 131.91, 130.52, 129.09, 127.97, 127.20, 127.12, 126.60, 124.71, 124.61, 121.71, 51.41, 29.53, 25.16, 20.45, 14.68.

*Methyl 3-(5-bromo-4-(4-n-propylnaphth-1-yl)-4H-1,2,4-triazol-3-yl)propionate* (**9i**): White solid; 1.57 g (65%); m.p. 104.5–106 °C. ^1^H-NMR (DMSO-*d*_6_, 400 MHz) δ: 8.27 (d, *J* = 8.4 Hz, 1H), 7.57–7.71 (m, 4H), 7.10 (d, *J* = 8.4 Hz, 1H), 3.51 (s, 3H), 3.13 (t, *J* = 7.6 Hz, 2H), 2.63–2.72 (m, 3H), 2.52–2.57 (m, 1H), 1.74–1.80 (m, 2H), 1.02 (t, *J* = 7.4 Hz, 3H). ^13^C-NMR (DMSO-*d*_6_, 100 MHz) δ: 171.80, 156.43, 142.09, 132.06, 130.52, 129.15, 127.93, 127.14, 126.42, 125.65, 124.88, 121.69, 51.41, 34.19, 29.51, 23.41, 20.44, 13.97.

*Methyl 3-(5-bromo-4-(4-isopropylnaphth-1-yl)-4H-1,2,4-triazol-3-yl)propionate* (**9j**): White solid; 1.50 g (62%); m.p. 108.5–110 °C. ^1^H-NMR (DMSO-*d*_6_, 400 MHz) δ: 8.36 (d, *J* = 8.4 Hz, 1H), 7.61–7.72 (m, 4H), 7.11 (d, *J* = 8.4 Hz, 1H), 3.83–3.90 (m, 1H), 3.52 (s, 3H), 2.69–2.74 (m, 2H), 2.63–2.66 (m, 1H), 2.47–2.54 (m, 1H), 1.40 (d, *J* = 6.8 Hz, 6H). ^13^C-NMR (DMSO-*d*_6_, 100 MHz) δ: 171.81, 156.44, 147.83, 131.48, 130.52, 129.14, 127.82, 127.17, 126.98, 126.59, 124.27, 121.78, 51.40, 29.47, 28.16, 23.28, 23.22, 20.45.

*Methyl 3-(5-bromo-4-(4-methoxynaphth-1-yl)-4H-1,2,4-triazol-3-yl)propionate* (**9k**): White solid; 0.70 g (30%); m.p. 124.5–126.5 °C. ^1^H-NMR (DMSO-*d*_6_, 400 MHz) δ: 8.29–8.31 (m, 1H), 7.68 (d, *J* = 8.4 Hz, 1H), 7.62–7.66 (m, 2H), 7.17 (d, *J* = 8.4 Hz, 1H), 7.03–7.05 (m, 1H), 4.08 (s, 3H), 3.52 (s, 3H), 2.66–2.72 (m, 3H), 2.51–2.58 (m, 1H). ^13^C-NMR (DMSO-*d*_6_, 100 MHz) δ: 171.80, 156.61, 156.52, 130.90, 129.75, 128.77, 127.66, 126.57, 125.15, 122.41, 121.12, 121.01, 104.11, 56.13, 51.41, 29.59, 20.44.

*Methyl 3-(5-bromo-4-(4-ethoxynaphth-1-yl)-4H-1,2,4-triazol-3-yl)propionate* (**9l**): White solid; 0.75 g (31%); m.p. 120–122.5 °C. ^1^H-NMR (DMSO-*d*_6_, 400 MHz) δ: 8.31–8.33 (m, 1H), 7.63–7.66 (m, 3H), 7.14 (d, *J* = 8.4 Hz, 1H), 7.02–7.04 (m, 1H), 4.33 (q, *J* = 6.9 Hz, 2H), 3.52 (s, 3H), 2.66–2.72 (m, 3H), 2.51–2.58 (m, 1H), 1.51 (t, *J* = 7.0 Hz, 3H). ^13^C-NMR (DMSO-*d*_6_, 100 MHz) δ: 171.78, 156.60, 155.77, 130.90, 129.79, 128.72, 127.66, 126.47, 125.22, 122.48, 120.96, 120.90, 104.66, 64.14, 51.40, 29.58, 20.43, 14.46.

*Methyl 3-(5-bromo-4-(4-bromonaphth-1-yl)-4H-1,2,4-triazol-3-yl)propionate* (**9m**): White solid; 1.61 g (61%); m.p. 162–163.5 °C. ^1^H-NMR (DMSO-*d*_6_, 400 MHz) δ: 8.32 (d, *J* = 8.4 Hz, 1H), 8.16 (d, *J* = 8.0 Hz, 1H), 7.85 (t, *J* = 7.6 Hz, 1H), 7.72–7.78 (m, 2H), 7.19 (d, *J* = 8.4 Hz, 1H), 3.52 (s, 3H), 2.66–2.73 (m, 3H), 2.52–2.60 (m, 1H). ^13^C-NMR (DMSO-*d*_6_, 100 MHz) δ: 171.75, 156.37, 131.82, 130.18, 130.09, 130.02, 129.52, 129.21, 128.94, 127.70, 127.42, 124.94, 122.08, 51.42, 29.57, 20.40.

*Methyl 3-(5-bromo-4-((4-bromonaphth-1-yl)methyl)-4H-1,2,4-triazol-3-yl)propionate* (**9n**): White solid; 1.71 g (63%); m.p. 90.5–91.5 °C. ^1^H-NMR (DMSO-*d*_6_, 400 MHz) δ: 8.22–8.26 (m, 2H), 7.78–7.83 (m, 3H), 6.36 (d, *J* = 8.0 Hz, 1H), 5.78 (s, 2H), 3.55 (s, 3H), 2.90 (t, *J* = 7.0 Hz, 2H), 2.77 (t, *J* = 7.0 Hz, 2H). ^13^C-NMR (DMSO-*d*_6_, 100 MHz) δ: 171.96, 156.06, 131.25, 131.04, 130.82, 130.10, 129.68, 128.29, 127.75, 127.17, 123.78, 122.46, 121.80, 51.42, 44.94, 29.59, 20.13.

*Methyl 3-(5-bromo-4-(2-(4-bromonaphth-1-yl)ethyl)-4H-1,2,4-triazol-3-yl)propionate* (**9o**): White foam; 1.82 g (65%). ^1^H-NMR (DMSO-*d*_6_, 400 MHz) δ: 8.18–8.21 (m, 1H), 8.14–8.16 (m, 1H), 7.77 (d, *J* = 8.0 Hz, 1H), 7.66–7.74 (m, 2H), 7.09 (d, *J* = 7.6 Hz, 1H), 4.26 (t, *J* = 7.0 Hz, 2H), 3.57 (s, 3H), 3.46 (t, *J* = 7.0 Hz, 2H), 2.60–2.68 (m, 4H). ^13^C-NMR (DMSO-*d*_6_, 100 MHz) δ: 172.00, 155.20, 133.79, 132.74, 131.28, 129.59, 128.89, 127.99, 127.69, 127.47, 127.21, 124.05, 121.28, 51.44, 44.66, 31.64, 29.62, 19.78.

*Methyl 4-(5-bromo-4-((4-bromonaphth-1-yl)methyl)-4H-1,2,4-triazol-3-yl)butanoate* (**9p**): White solid; 1.77 g (63%); m.p. 154–155.5 °C. ^1^H-NMR (DMSO-*d*_6_, 400 MHz) δ: 8.21–8.25 (m, 2H), 7.78–7.82 (m, 3H), 6.31 (d, *J* = 8.0 Hz, 1H), 5.74 (s, 2H), 3.49 (s, 3H), 2.71 (t, *J* = 7.4 Hz, 2H), 2.36 (t, *J* = 7.2 Hz, 2H), 1.81–1.89 (m, 2H). ^13^C-NMR (DMSO-*d*_6_, 100 MHz) δ: 172.76, 156.64, 131.43, 131.04, 130.78, 129.98, 129.70, 128.28, 127.73, 127.17, 123.72, 122.31, 121.76, 51.16, 44.99, 32.15, 23.72, 21.52.

*Methyl 5-(5-bromo-4-((4-bromonaphth-1-yl)methyl)-4H-1,2,4-triazol-3-yl)pentanoate* (**9q**): Colorless oil; 1.76 g (61%). ^1^H-NMR (DMSO-*d*_6_, 400 MHz) δ: 8.21–8.25 (m, 2H), 7.77–7.82 (m, 3H), 6.31 (d, *J* = 8.0 Hz, 1H), 5.74 (s, 2H), 3.50 (s, 3H), 2.68 (t, *J* = 7.2 Hz, 2H), 2.22 (t, *J* = 7.2 Hz, 2H), 1.49–1.60 (m, 4H). ^13^C-NMR (DMSO-*d*_6_, 100 MHz) δ: 173.04, 156.96, 131.53, 131.06, 130.81, 129.87, 129.69, 128.30, 127.75, 127.18, 123.76, 122.38, 121.76, 51.10, 44.98, 32.73, 25.48, 24.11, 23.68.

#### 3.2.13. General Procedure for the Synthesis of **9d** and **9e**

To a stirred solution of MeONa (150 mmol) in dried MeOH (for **9d**) or EtONa (150 mmol) in dried EtOH (for **9e**) prepared by dissolving Na (3.45 g, 150 mmol) in dried MeOH (100 mL) or EtOH (100 mL) was added **9b** (12.01 g, 30 mmol). The resulting mixture was stirred at reflux under N_2_ until the completion of reaction as indicated by TLC analysis (typically within 24 h).

On cooling to room temperature, the reaction mixture was poured into ice-water (200 mL) and the aqueous mixture thus obtained was extracted immediately with CH_2_Cl_2_ (50 mL × 3). The combined extracts were washed with 5% brine (50 mL), dried over anhydrous Na_2_SO_4_, and evaporated on a rotary evaporator to afford a residue, which was purified by column chromatography to produce **9d** or **9e** after trituration with EtOAc/*n*-hexane.

*Methyl 3-(4-(4-cyclopropylnaphth-1-yl)-5-methoxy-4H-1,2,4-triazol-3-yl)propionate* (**9d**): White solid; 1.48 g (14%); m.p. 115–116.5 °C. ^1^H-NMR (DMSO-*d*_6_, 400 MHz) δ: 8.54 (d, *J* = 8.4 Hz, 1H), 7.71 (t, *J* = 7.6 Hz, 1H), 7.63 (t, *J* = 7.6 Hz, 1H), 7.54 (d, *J* = 7.2 Hz, 1H), 7.38 (d, *J* = 7.6 Hz, 1H), 7.23 (d, *J* = 8.4 Hz, 1H), 3.91 (s, 3H), 3.51 (s, 3H), 2.64 (t, *J* = 7.0 Hz, 2H), 2.51–2.60 (m, 2H), 2.38–2.47 (m, 1H), 1.10–1.13 (m, 2H), 0.77–0.87 (m, 2H). ^13^C-NMR (DMSO-*d*_6_, 100 MHz) δ: 171.95, 159.33, 150.97, 141.82, 133.45, 129.14, 127.74, 126.96, 126.39, 126.10, 124.98, 122.73, 121.83, 57.47, 51.34, 29.47, 20.19, 12.82, 7.17, 6.88.

*Ethyl 3-(4-(4-cyclopropylnaphth-1-yl)-5-ethoxy-4H-1,2,4-triazol-3-yl)propionate* (**9e**): White solid; 1.48 g (13%); m.p. 94.5–96 °C. ^1^H-NMR (DMSO-*d*_6_, 400 MHz) δ: 8.54 (d, *J* = 8.4 Hz, 1H), 7.71 (t, *J* = 7.0 Hz, 1H), 7.63 (t, *J* = 7.2 Hz, 1H), 7.53 (d, *J* = 7.6 Hz, 1H), 7.38 (d, *J* = 7.6 Hz, 1H), 7.23 (d, *J* = 8.4 Hz, 1H), 4.31–4.37 (m, 2H), 3.96 (q, *J* = 7.1 Hz, 2H), 2.56–2.62 (m, 2H), 2.49–2.54 (m, 2H), 2.38–2.44 (m, 1H), 1.08–1.18 (m, 8H), 0.81–0.84 (m, 2H). ^13^C-NMR (DMSO-*d*_6_, 100 MHz) δ: 171.48, 158.63, 150.70, 141.72, 133.45, 129.14, 127.65, 126.95, 126.51, 126.03, 124.97, 122.75, 121.87, 66.42, 59.91, 29.63, 20.21, 14.22, 13.95, 12.81, 7.11, 6.95.

#### 3.2.14. General Procedure for the Synthesis of **10a**–**10q**

To a stirred solution of **9a**–**9q** (1.5 mmol) in EtOH (15 mL) was added an aqueous solution of LiOH prepared by dissolving LiOH·H_2_O (0.19 g, 4.5 mmol) in water (1 mL), and the resulting mixture was stirred at room temperature until the completion of reaction as indicated by TLC analysis (typically within 3 h).

The reaction mixture was poured into ice-water (100 mL) and the mixture thus obtained was acidified (pH = 1–2) by concentrated hydrochloric acid and extracted with CH_2_Cl_2_ (50 mL × 3). The combined extracts were washed with water (50 mL), dried over anhydrous Na_2_SO_4_, and evaporated on a rotary evaporator to afford a residue, which was purified by column chromatography to produce **10a**–**10q** after trituration with EtOAc/*n*-hexane.

*3-(5-Chloro-4-(4-cyclopropylnaphth-1-yl)-4H-1,2,4-triazol-3-yl)propionic acid* (**10a**): White solid; 0.45 g (88%); m.p. 146–147.5 °C. ^1^H-NMR (DMSO-*d*_6_, 400 MHz) δ: 12.21 (brs, 1H), 8.58 (d, *J* = 8.4 Hz, 1H), 7.72–7.76 (m, 1H), 7.63–7.67 (m, 2H), 7.44 (d, *J* = 7.6 Hz, 1H), 7.16 (d, *J* = 8.0 Hz, 1H), 2.44–2.65 (m, 5H), 1.13–1.16 (m, 2H), 0.86–0.87 (m, 2H). ^13^C-NMR (DMSO-*d*_6_, 100 MHz) δ: 172.81, 156.22, 142.79, 141.38, 133.45, 128.73, 128.16, 127.22, 126.49, 126.21, 125.15, 122.70, 121.50, 29.69, 20.58, 12.86, 7.23, 7.16.

*3-(5-Bromo-4-(4-cyclopropylnaphth-1-yl)-4H-1,2,4-triazol-3-yl)propionic acid* (**10b**): White solid; 0.52 g (89%); m.p. 132.5–134.5 °C. ^1^H-NMR (DMSO-*d*_6_, 400 MHz) δ: 12.21 (brs, 1H), 8.58 (d, *J* = 8.4 Hz, 1H), 7.73 (t, *J* = 7.6 Hz, 1H), 7.61–7.66 (m, 2H), 7.44 (d, *J* = 7.6 Hz, 1H), 7.11 (d, *J* = 8.4 Hz, 1H), 2.42–2.65 (m, 5H), 1.12–1.16 (m, 2H), 0.84-0.88 (m, 2H). ^13^C-NMR (DMSO-*d*_6_, 100 MHz) δ: 172.85, 156.70, 142.66, 133.46, 130.42, 128.87, 128.09, 127.20, 127.11, 126.55, 125.13, 122.72, 121.68, 29.84, 20.58, 12.89, 7.24, 7.19.

*3-(4-(4-Cyclopropylnaphth-1-yl)-5-iodo-4H-1,2,4-triazol-3-yl)propionic acid* (**10c**): White solid; 0.55 g (85%); m.p. 151 °C (dec). ^1^H-NMR (DMSO-*d*_6_, 400 MHz) δ: 12.18 (brs, 1H), 8.57 (d, *J* = 8.4 Hz, 1H), 7.72 (t, *J* = 7.4 Hz, 1H), 7.62 (t, *J* = 7.6 Hz, 1H), 7.54 (d, *J* = 7.6 Hz, 1H), 7.43 (d, *J* = 7.6 Hz, 1H), 7.03 (d, *J* = 8.4 Hz, 1H), 2.46–2.66 (m, 5H), 1.12–1.18 (m, 2H), 0.86–0.90 (m, 2H). ^13^C-NMR (DMSO-*d*_6_, 100 MHz) δ: 172.84, 156.52, 142.34, 133.41, 129.08, 128.49, 127.87, 127.11, 126.61, 125.04, 122.66, 121.94, 106.11, 30.13, 20.45, 12.86, 7.26, 7.15.

*3-(4-(4-Cyclopropylnaphth-1-yl)-5-methoxy-4H-1,2,4-triazol-3-yl)propionic acid* (**10d**): White solid; 0.46 g (90%); m.p. 167 °C (dec). ^1^H-NMR (DMSO-*d*_6_, 400 MHz) δ: 12.18 (brs, 1H), 8.54 (d, *J* = 8.4 Hz, 1H), 7.70 (t, *J* = 7.2 Hz, 1H), 7.62 (t, *J* = 7.4 Hz, 1H), 7.53 (d, *J* = 7.6 Hz, 1H), 7.38 (d, *J* = 7.6 Hz, 1H), 7.23 (d, *J* = 8.4 Hz, 1H), 3.90 (s, 3H), 2.47–2.57 (m, 4H), 2.34–2.43 (m, 1H), 1.08–1.16 (m, 2H), 0.79–0.87 (m, 2H). ^13^C-NMR (DMSO-*d*_6_, 100 MHz) δ: 173.02, 159.30, 151.22, 141.77, 133.45, 129.16, 127.71, 126.94, 126.47, 126.09, 124.97, 122.75, 121.88, 57.45, 29.87, 20.35, 12.83, 7.14, 6.88.

*3-(4-(4-Cyclopropylnaphth-1-yl)-5-ethoxy-4H-1,2,4-triazol-3-yl)propionic acid* (**10e**): White solid; 0.46 g (87%); m.p. 154.5–155.5 °C. ^1^H-NMR (DMSO-*d*_6_, 400 MHz) δ: 12.13 (brs, 1H), 8.54 (d, *J* = 8.4 Hz, 1H), 7.70 (t, *J* = 7.4 Hz, 1H), 7.62 (t, *J* = 7.6 Hz, 1H), 7.52 (d, *J* = 7.6 Hz, 1H), 7.38 (d, *J* = 7.6 Hz, 1H), 7.23 (d, *J* = 8.4 Hz, 1H), 4.30–4.38 (m, 2H), 2.46–2.57 (m, 4H), 2.32–2.41 (m, 1H), 1.11–1.18 (m, 5H), 0.82–0.87 (m, 2H). ^13^C-NMR (DMSO-*d*_6_, 100 MHz) δ: 173.00, 158.63, 150.92, 141.70, 133.46, 129.15, 127.64, 126.95, 126.57, 126.05, 124.97, 122.77, 121.93, 66.42, 29.77, 20.33, 14.23, 12.83, 7.10, 6.96.

*3-(5-Bromo-4-(naphth-1-yl)-4H-1,2,4-triazol-3-yl)propionic acid* (**10f**): White solid; 0.46 g (88%); m.p. 177.5–179 °C. ^1^H-NMR (DMSO-*d*_6_, 400 MHz) δ: 12.22 (brs, 1H), 8.24 (t, *J* = 4.6 Hz, 1H), 8.15 (d, *J* = 7.6 Hz, 1H), 7.61–7.74 (m, 4H), 7.14 (d, *J* = 8.0 Hz, 1H), 2.61–2.68 (m, 3H), 2.46–2.55 (m, 1H). ^13^C-NMR (DMSO-*d*_6_, 100 MHz) δ: 172.80, 156.60, 133.78, 130.98, 130.20, 128.92, 128.86, 128.67, 128.44, 127.29, 126.92, 125.82, 121.11, 29.84, 20.56.

*3-(5-Bromo-4-(4-methylnaphth-1-yl)-4H-1,2,4-triazol-3-yl)propionic acid* (**10g**): White solid; 0.46 g (86%); m.p. 186 °C (dec). ^1^H-NMR (DMSO-*d*_6_, 400 MHz) δ: 12.24 (brs, 1H), 8.20 (d, *J* = 8.4 Hz, 1H), 7.68–7.72 (m, 1H), 7.57–7.65 (m, 3H), 7.11 (d, *J* = 8.4 Hz, 1H), 2.76 (s, 3H), 2.59–2.64 (m, 3H), 2.46–2.53 (m, 1H). ^13^C-NMR (DMSO-*d*_6_, 100 MHz) δ: 172.96, 156.75, 137.99, 132.68, 130.41, 128.92, 128.08, 127.21, 127.18, 126.50, 126.28, 125.22, 121.60, 30.07, 20.65, 19.10.

*3-(5-Bromo-4-(4-ethylnaphth-1-yl)-4H-1,2,4-triazol-3-yl)propionic acid* (**10h**): White solid; 0.48 g (85%); m.p. 167.5–170 °C. ^1^H-NMR (DMSO-*d*_6_, 400 MHz) δ: 12.21 (brs, 1H), 8.27 (d, *J* = 8.4 Hz, 1H), 7.58–7.72 (m, 4H), 7.11 (d, *J* = 8.4 Hz, 1H), 3.19 (q, *J* = 7.5 Hz, 2H), 2.58–2.67 (m, 2H), 2.45–2.53 (m, 2H), 1.37 (t, *J* = 7.6 Hz, 3H). ^13^C-NMR (DMSO-*d*_6_, 100 MHz) δ: 172.87, 156.73, 143.57, 131.95, 130.46, 129.15, 127.96, 127.22, 126.62, 124.72, 124.62, 121.82, 29.87, 25.20, 20.62, 14.69.

*3-(5-Bromo-4-(4-n-propylnaphth-1-yl)-4H-1,2,4-triazol-3-yl)propionic acid* (**10i**): White solid; 0.52 g (89%); m.p. 139.5–141.5 °C. ^1^H-NMR (DMSO-*d*_6_, 400 MHz) δ: 12.20 (brs, 1H), 8.26 (d, *J* = 8.4 Hz, 1H), 7.56–7.71 (m, 4H), 7.10 (d, *J* = 8.0 Hz, 1H), 3.13 (t, *J* = 7.6 Hz, 2H), 2.58–2.64 (m, 2H), 2.45–2.52 (m, 2H), 1.72–1.80 (m, 2H), 1.02 (t, *J* = 7.4 Hz, 3H). ^13^C-NMR (DMSO-*d*_6_, 100 MHz) δ: 173.52, 157.37, 142.76, 132.76, 131.13, 129.88, 128.61, 127.91, 127.83, 127.12, 126.34, 125.58, 122.46, 34.90, 30.52, 24.11, 21.27, 14.69.

*3-(5-Bromo-4-(4-isopropylnaphth-1-yl)-4H-1,2,4-triazol-3-yl)propionic acid* (**10j**): White solid; 0.48 g (83%); m.p. 197 °C (dec). ^1^H-NMR (DMSO-*d*_6_, 400 MHz) δ: 12.21 (brs, 1H), 8.36 (d, *J* = 8.4 Hz, 1H), 7.60–7.72 (m, 4H), 7.11 (d, *J* = 8.0 Hz, 1H), 3.83–3.90 (m, 1H), 2.59–2.65 (m, 3H), 2.44–2.51 (m, 1H), 1.40 (d, *J* = 6.8 Hz, 6H). ^13^C-NMR (DMSO-*d*_6_, 100 MHz) δ: 172.85, 156.69, 147.82, 131.49, 130.45, 129.17, 127.81, 127.18, 127.06, 126.59, 124.27, 121.86, 121.78, 29.79, 28.17, 23.31, 23.25, 20.58.

*3-(5-Bromo-4-(4-methoxynaphth-1-yl)-4H-1,2,4-triazol-3-yl)propionic acid* (**10k**): White solid; 0.49 g (87%); m.p. 188 °C (dec). ^1^H-NMR (DMSO-*d*_6_, 400 MHz) δ: 12.20 (brs, 1H), 8.29–8.31 (m, 1H), 7.67 (d, *J* = 8.0 Hz, 1H), 7.62–7.65 (m, 2H), 7.17 (d, *J* = 8.4 Hz, 1H), 7.03–7.06 (m, 1H), 4.08 (s, 3H), 2.59–2.66 (m, 3H), 2.47–2.52 (m, 1H). ^13^C-NMR (DMSO-*d*_6_, 100 MHz) δ: 172.83, 156.86, 156.51, 130.82, 129.77, 128.75, 127.66, 126.58, 125.16, 122.40, 121.19, 121.08, 104.13, 56.15, 29.90, 20.56.

*3-(5-Bromo-4-(4-ethoxynaphth-1-yl)-4H-1,2,4-triazol-3-yl)propionic acid* (**10l**): White solid; 0.52 g (89%); m.p. 108–110.5 °C. ^1^H-NMR (DMSO-*d*_6_, 400 MHz) δ: 12.20 (brs, 1H), 8.30–8.34 (m, 1H), 7.60–7.65 (m, 3H), 7.14 (d, *J* = 8.0 Hz, 1H), 7.01–7.05 (m, 1H), 4.33 (q, *J* = 6.9 Hz, 2H), 2.59–2.67 (m, 3H), 2.47–2.55 (m, 1H), 1.51 (t, *J* = 7.0 Hz, 3H). ^13^C-NMR (DMSO-*d*_6_, 100 MHz) δ: 172.81, 156.84, 155.75, 130.82, 129.81, 128.70, 127.66, 126.47, 125.22, 122.47, 121.03, 120.97, 104.66, 64.14, 29.89, 20.55, 14.47.

*3-(5-Bromo-4-(4-bromonaphth-1-yl)-4H-1,2,4-triazol-3-yl)propionic acid* (**10m**): White solid; 0.58 g (91%); m.p. 202 °C (dec). ^1^H-NMR (DMSO-*d*_6_, 400 MHz) δ: 12.21 (brs, 1H), 8.32 (d, *J* = 8.4 Hz, 1H), 8.15 (d, *J* = 8.0 Hz, 1H), 7.85 (t, *J* = 7.8 Hz, 1H), 7.75 (d, *J* = 7.6 Hz, 1H), 7.72 (d, *J* = 8.0 Hz, 1H), 7.19 (d, *J* = 8.4 Hz, 1H), 2.60–2.69 (m, 3H), 2.49–2.56 (m, 1H). ^13^C-NMR (DMSO-*d*_6_, 100 MHz) δ: 172.80, 156.63, 131.83, 130.11, 130.02, 129.50, 129.22, 129.02, 127.71, 127.42, 124.91, 122.17, 29.87, 20.52.

*3-(5-Bromo-4-((4-bromonaphth-1-yl)methyl)-4H-1,2,4-triazol-3-yl)propionic acid* (**10n**): White solid; 0.56 g (85%); m.p. 173–176 °C. ^1^H-NMR (DMSO-*d*_6_, 400 MHz) δ: 12.22 (brs, 1H), 8.22–8.26 (m, 2H), 7.76–7.82 (m, 3H), 6.36 (d, *J* = 8.0 Hz, 1H), 5.77 (s, 2H), 2.85 (t, *J* = 7.0 Hz, 2H), 2.69 (t, *J* = 7.2 Hz, 2H). ^13^C-NMR (DMSO-*d*_6_, 100 MHz) δ: 173.68, 157.03, 131.97, 131.74, 131.52, 130.70, 130.38, 128.99, 128.46, 127.88, 124.48, 123.21, 122.49, 45.63, 30.62, 20.93.

*3-(5-Bromo-4-(2-(4-bromonaphth-1-yl)ethyl)-4H-1,2,4-triazol-3-yl)propionic acid* (**10o**): White solid; 0.57 g (84%); m.p. 190–192 °C. ^1^H-NMR (DMSO-*d*_6_, 400 MHz) δ: 12.21 (brs, 1H), 8.15–8.20 (m, 2H), 7.76 (d, *J* = 7.6 Hz, 1H), 7.66–7.73 (m, 2H), 7.11 (d, *J* = 8.0 Hz, 1H), 4.26 (t, *J* = 7.0 Hz, 2H), 3.46 (t, *J* = 7.0 Hz, 2H), 2.71 (t, *J* = 6.6 Hz, 2H), 2.62 (t, *J* = 6.4 Hz, 2H). ^13^C-NMR (DMSO-*d*_6_, 100 MHz) δ: 173.10, 155.49, 133.82, 132.78, 131.28, 129.58, 128.82, 127.98, 127.68, 127.47, 127.22, 124.07, 121.26, 44.63, 31.63, 29.96, 19.93.

*4-(5-Bromo-4-((4-bromonaphth-1-yl)methyl)-4H-1,2,4-triazol-3-yl)butanoic acid* (**10p**): White solid; 0.56 g (83%); m.p. 177–179 °C. ^1^H-NMR (DMSO-*d*_6_, 400 MHz) δ: 12.02 (brs, 1H), 8.21–8.25 (m, 2H), 7.76–7.82 (m, 3H), 6.30 (d, *J* = 8.0 Hz, 1H), 5.74 (s, 2H), 2.71 (t, *J* = 7.4 Hz, 2H), 2.27 (t, *J* = 7.2 Hz, 2H), 1.78–1.86 (m, 2H). ^13^C-NMR (DMSO-*d*_6_, 100 MHz) δ: 173.91, 156.81, 131.47, 131.05, 130.79, 129.95, 129.71, 128.30, 127.75, 127.18, 123.74, 122.27, 121.75, 45.01, 32.49, 23.84, 21.67.

*5-(5-Bromo-4-((4-bromonaphth-1-yl)methyl)-4H-1,2,4-triazol-3-yl)pentanoic acid* (**10q**): White solid; 0.57 g (82%); m.p. 166.5–168 °C. ^1^H-NMR (DMSO-*d*_6_, 400 MHz) δ: 11.95 (brs, 1H), 8.21–8.25 (m, 2H), 7.76–7.83 (m, 3H), 6.31 (d, *J* = 8.0 Hz, 1H), 5.74 (s, 2H), 2.68 (t, *J* = 7.4 Hz, 2H), 2.14 (t, *J* = 7.2 Hz, 2H), 1.58–1.64 (m, 2H), 1.45–1.53 (m, 2H). ^13^C-NMR (DMSO-*d*_6_, 100 MHz) δ: 174.21, 157.03, 131.54, 131.07, 130.83, 129.84, 129.70, 128.30, 127.76, 127.19, 123.77, 122.39, 121.78, 44.99, 33.15, 25.57, 24.19, 23.79.

#### 3.2.15. General Procedure for the Synthesis of **1a**–**1r**

To a stirred mixture of **7a** or **10a**–**10q** (1 mmol, accurately weighted to four decimal places) in MeOH (5 mL) was added an aqueous solution of NaOH (prepared by dissolving 0.0400 g, 1 mmol of NaOH in a minimal volume of water), and the resulting mixture was stirred at room temperature until a clear solution was obtained (typically within 1 h).

The reaction mixture was filtered off and the filtrate was evaporated on a rotary evaporator to give rise to a residue, which was co-evaporated with CH_2_Cl_2_ (10 mL × 3) on a rotary evaporator and further dried in vacuo at room temperature to yield **1a**–**1r**.

*Sodium 3-(4-(4-cyclopropylnaphth-1-yl)-4H-1,2,4-triazol-3-yl)propionate* (**1a**): White solid; 0.32 g (98%); m.p. 140 °C (dec). ^1^H-NMR (MeOH-*d*_4_, 400 MHz) δ: 8.59 (d, *J* = 8.4 Hz, 1H), 8.56 (s, 1H), 7.68 (t, *J* = 7.6 Hz, 1H), 7.60 (t, *J* = 7.6 Hz, 1H), 7.55 (d, *J* = 7.6 Hz, 1H), 7.42 (d, *J* = 7.6 Hz, 1H), 7.19 (d, *J* = 7.6 Hz, 1H), 2.75–2.80 (m, 2H), 2.43–2.53 (m, 3H), 1.16–1.19 (m, 2H), 0.82–0.85 (m, 2H). ^13^C-NMR (MeOH-*d*_4_, 100 MHz) δ: 179.27, 157.30, 146.36, 144.16, 135.44, 130.90, 129.21, 129.11, 128.23, 126.56, 126.33, 124.00, 122.79, 35.53, 22.43, 14.13, 7.59, 7.56. ESI-HRMS [M − Na]^−^: (*m*/*z*) calcd. for C_18_H_16_N_3_O_2_: 306.1243, found: 306.1235.

*Sodium 3-(5-chloro-4-(4-cyclopropylnaphth-1-yl)-4H-1,2,4-triazol-3-yl)propionate* (**1b**): White solid; 0.36 g (99%); m.p. 135 °C (dec). ^1^H-NMR (MeOH-*d*_4_, 400 MHz) δ: 8.61 (d, *J* = 8.4 Hz, 1H), 7.67–7.72 (m, 1H), 7.60–7.64 (m, 1H), 7.57 (d, *J* = 7.6 Hz, 1H), 7.45 (dd, *J* = 0.6 Hz and 7.8 Hz, 1H), 7.17 (d, *J* = 8.4 Hz, 1H), 2.65–2.78 (m, 2H), 2.40–2.59 (m, 3H), 1.16 (m, 2H), 0.84–0.88 (m, 2H). ^13^C-NMR (MeOH-*d*_4_, 100 MHz) δ: 178.94, 159.53, 144.97, 143.92, 135.58, 130.72, 129.35, 128.33, 127.83, 127.67, 126.50, 124.12, 122.56, 34.80, 23.54, 14.16, 7.63. ESI-HRMS [M − Na]^−^: (*m*/*z*) calcd. for C_18_H_15_ClN_3_O_2_: 340.0853, found: 340.0854.

*Sodium 3-(5-bromo-4-(4-cyclopropylnaphth-1-yl)-4H-1,2,4-triazol-3-yl)propionate* (**1c**): White solid; 0.41 g (100%); m.p. 162 °C (dec). ^1^H-NMR (MeOH-*d*_4_, 400 MHz) δ: 8.61 (d, *J* = 8.8 Hz, 1H), 7.68 (t, *J* = 7.4 Hz, 1H), 7.60 (t, *J* = 7.4 Hz, 1H), 7.55 (d, *J* = 7.6 Hz, 1H), 7.44 (d, *J* = 7.6 Hz, 1H), 7.13 (d, *J* = 8.4 Hz, 1H), 2.68–2.77 (m, 2H), 2.38–2.57 (m, 3H), 1.14–1.20 (m, 2H), 0.83–0.89 (m, 2H). ^13^C-NMR (MeOH-*d*_4_, 100 MHz) δ: 178.07, 159.87, 144.91, 135.57, 130.78, 129.67, 129.28, 128.60, 128.32, 127.75, 126.47, 124.10, 122.72, 34.04, 23.17, 14.16, 7.65. ESI-HRMS [M − Na]^−^: (*m*/*z*) calcd. for C_18_H_15_BrN_3_O_2_: 384.0348 (^79^Br), found: 384.0339; calcd. for C_18_H_15_BrN_3_O_2_: 386.0327 (^81^Br), found: 386.0317.

*Sodium 3-(4-(4-cyclopropylnaphth-1-yl)-5-iodo-4H-1,2,4-triazol-3-yl)propionate* (**1d**): White solid; 0.44 g (97%); m.p. 158 °C (dec). ^1^H-NMR (MeOH-*d*_4_, 400 MHz) δ: 8.60 (d, *J* = 8.8 Hz, 1H), 7.68 (td, *J* = 0.9 Hz and 7.6 Hz, 1H), 7.57–7.61 (m, 1H), 7.50 (d, *J* = 7.6 Hz, 1H), 7.44 (d, *J* = 7.6 Hz, 1H), 7.07 (d, *J* = 8.4 Hz, 1H), 2.71–2.78 (m, 2H), 2.47–2.56 (m, 2H), 2.37–2.46 (m, 1H), 1.15–1.21 (m, 2H), 0.83-0.90 (m, 2H). ^13^C-NMR (MeOH-*d*_4_, 100 MHz) δ: 179.28, 160.15, 144.70, 135.57, 131.00, 129.99, 129.14, 128.27, 127.95, 126.43, 124.11, 122.99, 105.86, 35.45, 23.54, 14.18, 7.71, 7.60. ESI-HRMS [M − Na]^−^: (*m*/*z*) calcd. for C_18_H_15_IN_3_O_2_: 432.0209, found: 432.0200.

*Sodium 3-(4-(4-cyclopropylnaphth-1-yl)-5-methoxy-4H-1,2,4-triazol-3-yl)propionate* (**1e**): White solid; 0.36 g (99%); m.p. 133 °C (dec). ^1^H-NMR (MeOH-*d*_4_, 400 MHz) δ: 8.57 (d, *J* = 8.4 Hz, 1H), 7.66 (dt, *J* = 1.1 Hz and 7.7 Hz, 1H), 7.58 (dt, *J* = 1.2 Hz and 7.6 Hz, 1H), 7.48 (d, *J* = 7.6 Hz, 1H), 7.40 (d, *J* = 7.6 Hz, 1H), 7.25 (d, *J* = 8.4 Hz, 1H), 3.99 (s, 3H), 2.65 (t, *J* = 8.0 Hz, 2H), 2.43–2.51 (m, 2H), 2.31–2.39 (m, 1H), 1.14–1.18 (m, 2H), 0.81–0.84 (m, 2H). ^13^C-NMR (MeOH-*d*_4_, 100 MHz) δ: 179.46, 161.32, 155.00, 144.05, 135.55, 131.08, 128.87, 128.03, 127.80, 127.35, 126.32, 124.13, 122.86, 58.56, 35.19, 23.38, 14.12, 7.53, 7.44. ESI-HRMS [M − Na]^−^: (*m*/*z*) calcd. for C_19_H_18_N_3_O_3_: 336.1348, found: 336.1346.

*Sodium 3-(4-(4-cyclopropylnaphth-1-yl)-5-ethoxy-4H-1,2,4-triazol-3-yl)propionate* (**1f**): White solid; 0.37 g (98%); m.p. 101.5 °C (dec). ^1^H-NMR (MeOH-*d*_4_, 400 MHz) δ: 8.57 (d, *J* = 8.4 Hz, 1H), 7.66 (t, *J* = 7.0 Hz, 1H), 7.59 (t, *J* = 7.6 Hz, 1H), 7.48 (d, *J* = 7.6 Hz, 1H), 7.41 (d, *J* = 7.6 Hz, 1H), 7.26 (d, *J* = 8.0 Hz, 1H), 4.39 (q, *J* = 7.1 Hz, 2H), 2.64 (t, *J* = 7.6 Hz, 2H), 2.44–2.49 (m, 2H), 2.37–2.38 (m, 1H), 1.22 (t, *J* = 7.0 Hz, 3H), 1.14–1.19 (m, 2H), 0.82–0.86 (m, 2H). ^13^C-NMR (MeOH-*d*_4_, 100 MHz) δ: 178.85, 160.64, 154.61, 143.99, 135.57, 131.09, 128.82, 128.03, 127.89, 127.33, 126.33, 124.18, 122.93, 68.47, 34.74, 23.20, 14.63, 14.14, 7.51, 7.44. ESI-HRMS [M − Na]^−^: (*m*/*z*) calcd. for C_20_H_20_N_3_O_3_: 350.1505, found: 350.1497.

*Sodium 3-(5-bromo-4-(naphth-1-yl)-4H-1,2,4-triazol-3-yl)propionate* (**1g**): White solid; 0.37 g (100%); m.p. 182–184 °C. ^1^H-NMR (MeOH-*d*_4_, 400 MHz) δ: 8.18 (d, *J* = 7.6 Hz, 1H), 8.06–8.09 (m, 1H), 7.59–7.72 (m, 4H), 7.17–7.19 (m, 1H), 2.68–2.81 (m, 2H), 2.55–2.63 (m, 1H), 2.44–2.52 (m, 1H). ^13^C-NMR (MeOH-*d*_4_, 100 MHz) δ: 178.50, 159.84, 135.80, 132.52, 132.01, 130.82, 130.27, 129.92, 129.67, 128.52, 128.10, 126.72, 122.16, 34.33, 23.29. ESI-HRMS [M − Na]^−^: (*m*/*z*) calcd. for C_15_H_11_BrN_3_O_2_: 344.0035 (^79^Br), found: 344.0030; calcd. for C_15_H_11_BrN_3_O_2_: 346.0014 (^81^Br), found: 346.0010.

*Sodium 3-(5-bromo-4-(4-methylnaphth-1-yl)-4H-1,2,4-triazol-3-yl)propionate* (**1h**): White solid; 0.38 g (99%); m.p. 155 °C (dec). ^1^H-NMR (MeOH-*d*_4_, 400 MHz) δ: 8.21 (d, *J* = 8.4 Hz, 1H), 7.65–7.69 (m, 1H), 7.59–7.63 (m, 1H), 7.54 (s, 2H), 7.13–7.15 (m, 1H), 2.80 (s, 3H), 2.71–2.78 (m, 2H), 2.50–2.58 (m, 1H), 2.38–2.49 (m, 1H). ^13^C-NMR (MeOH-*d*_4_, 100 MHz) δ: 179.26, 160.14, 139.95, 134.69, 132.22, 130.82, 129.24, 128.69, 128.36, 127.74, 127.29, 126.28, 122.72, 35.11, 23.60, 19.64. ESI-HRMS [M − Na]^−^: (*m*/*z*) calcd. for C_16_H_13_BrN_3_O_2_: 358.0190 (^79^Br), found: 358.0192; calcd. for C_16_H_13_BrN_3_O_2_: 360.0171 (^81^Br), found: 360.0171.

*Sodium 3-(5-bromo-4-(4-ethylnaphth-1-yl)-4H-1,2,4-triazol-3-yl)propionate* (**1i**): White solid; 0.38 g (97%); m.p. 198 °C (dec). ^1^H-NMR (MeOH-*d*_4_, 400 MHz) δ: 8.27 (d, *J* = 8.4 Hz, 1H), 7.64–7.69 (m, 2H), 7.57–7.61 (m, 2H), 7.14 (d, *J* = 8.0 Hz, 1H), 3.23 (q, *J* = 7.6 Hz, 2H), 2.71–2.76 (m, 2H), 2.52–2.59 (m, 1H), 2.42–2.48 (m, 1H), 1.43 (t, *J* = 7.4 Hz, 3H). ^13^C-NMR (MeOH-*d*_4_, 100 MHz) δ: 179.13, 160.07, 145.72, 133.80, 132.18, 131.00, 129.11, 128.59, 128.32, 127.81, 125.88, 125.71, 122.82, 34.91, 26.90, 23.52, 15.37. ESI-HRMS [M − Na]^−^: (*m*/*z*) calcd. for C_17_H_15_BrN_3_O_2_: 372.0348 (^79^Br), found: 372.0349; calcd. for C_17_H_15_BrN_3_O_2_: 374.0327 (^81^Br), found: 374.0333.

*Sodium 3-(5-bromo-4-(4-n-propylnaphth-1-yl)-4H-1,2,4-triazol-3-yl)propionate* (**1j**): White solid; 0.41 g (99%); m.p. 183 °C (dec). ^1^H-NMR (MeOH-*d*_4_, 400 MHz) δ: 8.25 (d, *J* = 8.4 Hz, 1H), 7.64–7.68 (m, 1H), 7.53–7.61 (m, 3H), 7.14 (d, *J* = 8.0 Hz, 1H), 3.17 (t, *J* = 7.6 Hz, 2H), 2.67–2.76 (m, 2H), 2.54–2.62 (m, 1H), 2.43–2.51 (m, 1H), 1.79–1.88 (m, 2H), 1.08 (t, *J* = 7.4 Hz, 3H). ^13^C-NMR (MeOH-*d*_4_, 100 MHz) δ: 178.64, 159.97, 144.27, 134.04, 132.22, 131.11, 129.11, 128.65, 128.27, 127.64, 126.75, 126.07, 122.85, 36.06, 34.50, 25.06, 23.36, 14.47. ESI-HRMS [M − Na]^−^: (*m*/*z*) calcd. for C_18_H_17_BrN_3_O_2_: 386.0504 (^79^Br), found: 386.0509; calcd. for C_18_H_17_BrN_3_O_2_: 388.0484 (^81^Br), found: 388.0498.

*Sodium 3-(5-bromo-4-(4-isopropylnaphth-1-yl)-4H-1,2,4-triazol-3-yl)propionate* (**1k**): White solid; 0.41 g (100%); m.p. 194 °C (dec). ^1^H-NMR (MeOH-*d*_4_, 400 MHz) δ: 8.35 (d, *J* = 8.4 Hz, 1H), 7.57–7.69 (m, 4H), 7.14 (d, *J* = 8.4 Hz, 1H), 3.86-3.92 (m, 1H), 2.66-2.78 (m, 2H), 2.53–2.61 (m, 1H), 2.42–2.50 (m, 1H), 1.46 (d, *J* = 6.8 Hz, 3H), 1.46 (d, *J* = 6.8 Hz, 3H). ^13^C-NMR (MeOH-*d*_4_, 100 MHz) δ: 178.99, 160.08, 150.11, 133.54, 132.23, 131.13, 129.02, 128.48, 128.30, 127.84, 125.45, 122.95, 122.77, 34.82, 29.98, 23.83, 23.50. ESI-HRMS [M − Na]^−^: (*m*/*z*) calcd. for C_18_H_17_BrN_3_O_2_: 386.0504 (^79^Br), found: 386.0508; calcd. for C_18_H_17_BrN_3_O_2_: 388.0484 (^81^Br), found: 388.0494.

*Sodium 3-(5-bromo-4-(4-methoxynaphth-1-yl)-4H-1,2,4-triazol-3-yl)propionate* (**1l**): White solid; 0.39 g (98%); m.p. 211 °C (dec). ^1^H-NMR (MeOH-*d*_4_, 400 MHz) δ: 8.36-8.38 (m, 1H), 7.57–7.62 (m, 3H), 7.09 (d, *J* = 8.4 Hz, 1H), 7.05–7.07 (m, 1H), 4.11 (s, 3H), 2.72–2.77 (m, 2H), 2.53–2.61 (m, 1H), 2.44–2.50 (m, 1H). ^13^C-NMR (MeOH-*d*_4_, 100 MHz) δ: 178.60, 160.20, 158.96, 132.70, 131.71, 129.92, 128.86, 127.60, 127.38, 124.02, 122.59, 122.01, 104.54, 56.63, 34.57, 23.37. ESI-HRMS [M − Na]^−^: (*m*/*z*) calcd. for C_16_H_13_BrN_3_O_3_: 374.0140 (^79^Br), found: 374.0137; calcd. for C_16_H_13_BrN_3_O_3_: 376.0120 (^81^Br), found: 376.0115.

*Sodium 3-(5-bromo-4-(4-ethoxynaphth-1-yl)-4H-1,2,4-triazol-3-yl)propionate* (**1m**): White solid; 0.40 g (96%); m.p. 194 °C (dec). ^1^H-NMR (MeOH-*d*_4_, 400 MHz) δ: 8.38–8.41 (m, 1H), 7.58–7.61 (m, 3H), 7.05–7.07 (m, 2H), 4.33 (q, *J* = 7.1 Hz, 2H), 2.67–2.77 (m, 2H), 2.47–2.64 (m, 2H), 1.58 (t, *J* = 7.0 Hz, 3H). ^13^C-NMR (MeOH-*d*_4_, 100 MHz) δ: 178.02, 160.05, 158.23, 132.75, 131.73, 129.87, 128.84, 127.51, 127.45, 124.08, 122.33, 121.98, 105.17, 65.59, 33.99, 23.13, 14.97. ESI-HRMS [M − Na]^−^: (*m*/*z*) calcd. for C_17_H_15_BrN_3_O_3_: 388.0297 (^79^Br), found: 388.0299; calcd. for C_17_H_15_BrN_3_O_3_: 390.0276 (^81^Br), found: 390.0283.

*Sodium 3-(5-bromo-4-(4-bromonaphth-1-yl)-4H-1,2,4-triazol-3-yl)propionate* (**1n**): White solid; 0.43 g (97%); m.p. 204 °C (dec). ^1^H-NMR (MeOH-*d*_4_, 400 MHz) δ: 8.40 (d, *J* = 8.8 Hz, 1H), 8.05 (d, *J* = 7.6 Hz, 1H), 7.76–7.81 (m, 1H), 7.69–7.73 (m, 1H), 7.61 (d, *J* = 8.0 Hz, 1H), 7.22 (d, *J* = 8.4 Hz, 1H), 2.71–2.76 (m, 2H), 2.55–2.63 (m, 1H), 2.46–2.52 (m, 1H). ^13^C-NMR (MeOH-*d*_4_, 100 MHz) δ: 178.68, 159.89, 134.02, 131.96, 131.84, 131.03, 130.63, 130.38, 130.09, 129.09, 128.72, 127.02, 123.14, 34.54, 23.36. ESI-HRMS [M − Na]^−^: (*m*/*z*) calcd. for C_15_H_10_Br_2_N_3_O_2_: 423.9119, found: 423.9115.

*Sodium 3-(5-bromo-4-((4-bromonaphth-1-yl)methyl)-4H-1,2,4-triazol-3-yl)propionate* (**1o**): White solid; 0.46 g (100%); m.p. 152.5 °C (dec). ^1^H-NMR (MeOH-*d*_4_, 400 MHz) δ: 8.32–8.34 (m, 1H), 8.18–8.20 (m, 1H), 7.71–7.75 (m, 3H), 6.43 (d, *J* = 7.6 Hz, 1H), 5.84 (s, 2H), 2.94 (t, *J* = 7.4 Hz, 2H), 2.65 (t, *J* = 7.4 Hz, 2H). ^13^C-NMR (MeOH-*d*_4_, 100 MHz) δ: 178.62, 159.53, 133.22, 132.53, 131.92, 131.65, 130.71, 129.04, 128.94, 128.80, 124.18, 124.01, 123.78, 46.84, 34.65, 22.95. ESI-HRMS [M − Na]^−^: (*m*/*z*) calcd. for C_16_H_12_Br_2_N_3_O_2_: 437.9276, found: 437.9272.

*Sodium 3-(5-bromo-4-(2-(4-bromonaphth-1-yl)ethyl)-4H-1,2,4-triazol-3-yl)propionate* (**1p**): White solid; 0.47 g (99%); m.p. 167 °C (dec). ^1^H-NMR (MeOH-*d*_4_, 400 MHz) δ: 8.24–8.28 (m, 1H), 8.07–8.11 (m, 1H), 7.68 (d, *J* = 7.6 Hz, 1H), 7.61–7.66 (m, 2H), 7.07 (d, *J* = 7.6 Hz, 1H), 4.42 (t, *J* = 7.0 Hz, 2H), 3.54 (t, *J* = 6.8 Hz, 2H), 2.69 (t, *J* = 7.0 Hz, 2H), 2.57 (t, *J* = 7.2 Hz, 2H). ^13^C-NMR (MeOH-*d*_4_, 100 MHz) δ: 178.88, 158.84, 134.75, 134.50, 133.41, 130.81, 130.53, 129.16, 128.92, 128.60, 128.48, 124.85, 123.29, 46.60, 34.83, 33.24, 22.69. ESI-HRMS [M − Na]^−^: (*m*/*z*) calcd. for C_17_H_14_Br_2_N_3_O_2_: 451.9432, found: 451.9426.

*Sodium 4-(5-bromo-4-((4-bromonaphth-1-yl)methyl)-4H-1,2,4-triazol-3-yl)butanoate* (**1q**): White solid; 0.47 g (98%); m.p. 166 °C (dec). ^1^H-NMR (MeOH-*d*_4_, 400 MHz) δ: 8.30–8.33 (m, 1H), 8.16–8.19 (m, 1H), 7.71–7.75 (m, 3H), 6.39 (d, *J* = 8.0 Hz, 1H), 5.79 (s, 2H), 2.78 (t, *J* = 7.8 Hz, 2H), 2.21 (t, *J* = 7.2 Hz, 2H), 1.95–2.02 (m, 2H). ^13^C-NMR (MeOH-*d*_4_, 100 MHz) δ: 180.55, 159.43, 133.19, 132.49, 131.85, 131.78, 130.65, 129.05, 128.97, 128.91, 124.18, 124.04, 123.66, 46.83, 37.21, 25.94, 24.57. ESI-HRMS [M − Na]^−^: (*m*/*z*) calcd. for C_17_H_14_Br_2_N_3_O_2_: 451.9432, found: 451.9434.

*Sodium 5-(5-bromo-4-((4-bromonaphth-1-yl)methyl)-4H-1,2,4-triazol-3-yl)pentanoate* (**1r**): White solid; 0.48 g (98%); m.p. 146–149 °C. ^1^H-NMR (MeOH-*d*_4_, 400 MHz) δ: 8.32–8.34 (m, 1H), 8.16–8.19 (m, 1H), 7.71–7.76 (m, 3H), 6.42 (d, *J* = 8.0 Hz, 1H), 5.77 (s, 2H), 2.75 (t, *J* = 7.4 Hz, 2H), 2.11 (t, *J* = 7.4 Hz, 2H), 1.67–1.74 (m, 2H), 1.56–1.63 (m, 2H). ^13^C-NMR (MeOH-*d*_4_, 100 MHz) δ: 181.17, 159.48, 133.23, 132.52, 131.86, 131.78, 130.67, 129.09, 129.01, 128.98, 124.17, 124.13, 123.84, 46.83, 37.64, 27.66, 26.65, 26.05. ESI-HRMS [M-Na]^−^: (*m*/*z*) calcd. for C_18_H_16_Br_2_N_3_O_2_: 465.9589, found: 465.9588. 

### 3.3. In Vitro URAT1 Inhibitory Activity

The in vitro URAT1 inhibitory activity of the compounds **1a**–**1r** and lesinurad (in the form of sodium salt as well) was determined by the inhibition of URAT1-mediated [8-^14^C]uric acid uptake by human embryonic kidney 293 (HEK293) cells stably expressing human URAT1 (HEK293-URAT1 cells). Thus, HEK293-URAT1 cells were subcultured for 2–3 generations in DMEM (with 10% FBS, 100 IU/mL penicillin, and 50 μg/mL streptomycin at 37 °C, 5% CO_2_, and 95% air). After 48 h, when the HEK293-URAT1 cells were grown to confluence, the cells were treated by 0.25% trypsin for 1–2 min. Thereafter, the digested cells were diluted to a density of 1.5 × 10^5^/mL and inoculated into the 24-well cell culture plates for about 48 h. The HEK293-URAT1 cells were pre-incubated for 10 min in HBSS (Cl^−^ free) and then incubated in the uptake solution (PBS) containing 5 μM [8-^14^C]uric acid (2 μCi/mL) with or without the tested compounds (at a series of concentrations). The HEK293-URAT1 cells were then washed three times with ice-cold HBSS (Cl^−^ free) and treated by 0.1 M aqueous NaOH solution. After that, 500 μL of cell lysis was transferred to scintillation vials and solubilized with scintillation solution. The radioactivity in the cells was measured by a liquid scintillation counter (PerkinElmer, Waltham, MA, USA). The concentrations of the compounds to inhibit 50% [8-^14^C]uric acid uptake by HEK293 cells (IC_50_) were calculated with GraphPad Prism software (San Diego, CA, USA).

## 4. Conclusions

Systematic SAR exploration of a lesinurad-based hit **1c** led to the discovery of a highly potent URAT1 inhibitor, **1q**, which was 31-fold more potent than the parent lesinurad (IC_50_ = 0.23 μM against human URAT1 for **1q** vs. 7.18 μM for lesinurad). URAT1 inhibitor **1q** was structurally flexible as compared with parent lesinurad and could probably serve as a promising prototype scaffold for the further design of URAT1 inhibitors. The SAR study also demonstrated that the S atom in lesinurad was not indispensable for its URAT1-inhibitory activity. We will go on to perform some studies on some drug-like properties such as logP and solubility, and further SAR exploration of the naphthalene ring.

## Figures and Tables

**Table 1:** In vitro inhibitory activity of **1a**–**1r** as well as lesinurad against human URAT1 (IC_50_).

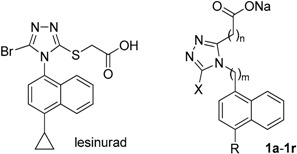					
**Compound**	**R**	**X**	**m**	**n**	**IC_50_ (μM)** ^a^
lesinurad	-	-	-	-	7.18 ± 1.13 ^b^
**1a**	cyclopropyl	H	0	2	>100
**1b**	cyclopropyl	Cl	0	2	6.3 ± 0.74
**1c**	cyclopropyl	Br	0	2	2.4 ± 0.15
**1d**	cyclopropyl	I	0	2	8.8 ± 1.0
**1e**	cyclopropyl	OMe	0	2	>100
**1f**	cyclopropyl	OEt	0	2	9.1 ± 1.2
**1g**	H	Br	0	2	10.5 ± 1.4
**1h**	Me	Br	0	2	12.6 ± 1.6
**1i**	Et	Br	0	2	8.4 ± 0.98
**1j**	*n*-Pr	Br	0	2	8.3 ± 1.1
**1k**	*i*-Pr	Br	0	2	1.1 ± 0.21
**1l**	OMe	Br	0	2	36.6 ± 5.3
**1m**	OEt	Br	0	2	11.2 ± 1.3
**1n**	Br	Br	0	2	0.85 ± 0.12
**1o**	Br	Br	1	2	0.61 ± 0.07
**1p**	Br	Br	2	2	34.7 ± 4.9
**1q**	Br	Br	1	3	0.23 ± 0.05
**1r**	Br	Br	1	4	1.24 ± 0.17

^a^ All the experiments were performed in triplicate, and the IC_50_ values were expressed as mean ± SD. ^b^ Reported value for lesinurad: IC_50_ = 7.3 μM against human URAT1 [19].

## References

[1] Punzi L., Scanu A., Ramonda R., Oliviero F. (2012). Gout as autoinflammatory disease: New mechanisms for more appropriated treatment targets. Autoimmun. Rev..

[2] Richette P., Bardin T. (2010). Gout. Lancet.

[3] Abeles A.M. (2015). Hyperuricemia, gout, and cardiovascular disease: An update. Curr. Rheumatol. Rep..

[4] Gustafsson D., Unwin R. (2013). The pathophysiology of hyperuricaemia and its possible relationship to cardiovascular disease, morbidity and mortality. BMC Nephrol..

[5] Liebman S.E., Taylor J.G., Bushinsky D.A. (2007). Uric acid nephrolithiasis. Curr. Rheumatol. Rep..

[6] Nuki G., Simkin P.A. (2006). A concise history of gout and hyperuricemia and their treatment. Arthritis Res. Ther..

[7] Kuo C.-F., Grainge M.J., Zhang W., Doherty M. (2015). Global epidemiology of gout: Prevalence, incidence and risk factors. Nat. Rev. Rheumatol..

[8] Zhu Y., Pandya B.J., Choi H.K. (2011). Prevalence of gout and hyperuricemia in the US general population: The national health and nutrition examination survey 2007–2008. Arthritis Rheum..

[9] Liu H., Zhang X.-M., Wang L.-Y., Liu B.-C. (2014). Prevalence of hyperuricemia among Chinese adults: A national cross-sectional survey using multistage, stratified sampling. J. Nephrol..

[10] Smith E., Hoy D., Cross M., Merriman T.R., Vos T., Buchbinder R., Woolf A., March L. (2014). The global burden of gout: Estimates from the global burden of disease 2010 study. Ann. Rheum. Dis..

[11] Wertheimer A., Morlock R., Becker M.A. (2013). A revised estimate of the burden of illness of gout. Curr. Ther. Res..

[12] Bernal J.A., Quilis N., Andrés M., Sivera F., Pascual E. (2016). Gout: Optimizing treatment to achieve a disease cure. Ther. Adv. Chronic. Dis..

[13] Gliozzi M., Malara N., Muscoli S., Mollace V. (2016). The treatment of hyperuricemia. Int. J. Cardiol..

[14] Singh J.A. (2012). Emerging therapies for gout. Expert Opin. Emerg. Drugs.

[15] Shahid H., Singh J.A. (2015). Investigational drugs for hyperuricemia. Expert Opin. Investig. Drugs.

[16] Burns C.M., Wortmann R.L. (2011). Gout therapeutics: New drugs for an old disease. Lancet.

[17] Enomoto A., Kimura H., Chairoungdua A., Shigeta Y., Jutabha P., Cha S.H., Hosoyamada M., Takeda M., Sekine T., Igarashi T. (2002). Molecular identification of a renal urate anion exchanger that regulates blood urate levels. Nature.

[18] Hyndman D., Liu S., Miner J.N. (2016). Urate handling in the human body. Curr. Rheumatol. Rep..

[19] Hoy S.M. (2016). Lesinurad: First global approval. Drugs.

[20] Stocks M.J., Cheshire D.R., Reynolds R. (2004). Efficient and regiospecific one-pot synthesis of substituted 1,2,4-triazoles. Org. Lett..

[21] Wender P.A., Koehler M.F.T., Sendzik M. (2003). A new synthetic approach to the C ring of known as well as novel bryostatin analogues. Org. Lett..

[22] Bal B.S., Childers W.E., Pinnick H.W. (1981). Oxidation of α,β-unsaturated aldehydes. Tetrahedron.

[23] Takahashi B., Funami H., Iwaki T., Maruoka H., Shibata M., Koyama M., Nagahira A., Kamiide Y., Kanki S., Igawa Y. (2015). Orally active ghrelin receptor inverse agonists and their actions on a rat obesity model. Bioorg. Med. Chem..

[24] Wu G., Deng Y., Wu C., Zhang Y., Wang J. (2014). Synthesis of α-aryl esters and nitriles: Deaminative coupling of α-aminoesters and α-aminoacetonitriles with arylboronic acids. Angew. Chem. Int. Ed..

[25] Nambo M., Yar M., Smith J.D., Crudden C.M. (2015). The concise synthesis of unsymmetric triarylacetonitriles via Pd-catalyzed sequential arylation: A new synthetic approach to tri- and tetraarylmethanes. Org. Lett..

[26] Zhang X., Wu J., Liu W., Liu Y., Xie Y., Shang Q., Zhou Z., Xu W., Tang L., Wang J. (2016). Discovery of flexible naphthyltriazolylmethane-based thioacetic acids as highly active uric acid transporter 1 (URAT1) inhibitors for the treatment of hyperuricemia of gout. Med. Chem..

[27] Zhao M.M., Li J., Mano E., Song Z.J., Tschaen D.M. (2005). Oxidation of primary alcohols to carboxylic acids with sodium chlorite catalyzed by tempo and bleach: 4-methoxyphenylacetic acid. Org. Synth..

[28] Sun H., Hu W., Wang Z.-H., Li Y.-Z., Zhao G.-L., Wang J.-W. (2008). Synthesis of two modified intermediates in epothilone biosynthesis. Chin. J. Org. Chem..

[29] Gould E., Lebl T., Slawin A.M.Z., Reid M., Smith A.D. (2010). Structural effects in yrazolidinone-mediated organocatalytic Dielse-Alder reactions. Tetrahedron.

[30] Hoffman A. (1929). Beta-phenylisobutylmethyl ketone and its derivatives. The synthesis of alpha- and of beta-phenylisovalerianic acid. J. Am. Chem. Soc..

[31] Morris D.J., Hayes A.M., Wills M. (2006). The “reverse-tethered” Ruthenium (II) catalyst for asymmetric transfer hydrogenation: Further applications. J. Org. Chem..

[32] Gallant M., Chauret N., Claveau D., Day S., Deschênes D., Dubé D., Huang Z., Lacombe P., Laliberté F., Lévesque J.-F. (2008). Design, synthesis, and biological evaluation of 8-biarylquinolines: A novel class of PDE4 inhibitors. Bioorg. Med. Chem. Lett..

[33] Beck A.L., Mascal M., Moody C.J., Coates W.J. (1992). Synthesis of 3,4-bridged indoles by photocyclisation reactions. Part 2. Photocyclisation of halogenoacetyl tryptophol derivatives and α-chloro indol-3-ylalkanoate esters. J. Chem. Soc. Perkin Trans. 1.

[34] Futatsugi K., Kung D.W., Orr S.T., Cabral S., Hepworth D., Aspnes G., Bader S., Bian J., Boehm M., Carpino P.A. (2015). Discovery and optimization of imidazopyridine-based inhibitors of diacylglycerol acyltransferase 2 (DGAT2). J. Med. Chem..

[35] Ishii Y., Yoshida T., Yamawaki K., Ogawa M. (1988). Lactone synthesis by a,ω-diols with hydrogen peroxide catalyzed by heteropoly acids combined with cetylpyridinium chloride. J. Org. Chem..

[36] Little R.D., Muller G.W., Venegas M.G., Carroll G.L., Bukhari A., Stone K. (1981). 1,3-Diyl trapping reactions: Fundamental investigations with application to the synthesis of linearly fused tricyclopentanoids. Tetrahedron.

[37] Gilchrist T.L., Richards P. (1983). Intramolecular cycloaddition reactions of in situ generated azoalkenes: Synthesis of pyrrolo[1,2-*b*]pyridazine derivatives from α-haloketone 4-pentenotlhydrazones. Synthesis.

[38] McElvain S.M., McKay G.R. (1955). Ketene acetals. XXXV. Cyclic ketene acetals and orthoesters from 2,2-dimethoxy-2,3-dihydropyran. J. Am. Chem. Soc..

[39] Huisgen R., Ott H. (1959). Die konfiguration der carbonestergruppe und die sondereigenschaften der lactone. Tetrahedron.

[40] Rink M., Krebber D., Fanslau D., Mehta S. (1966). Synthese von durchhydrierten dipiperidazino-tetrazin, -tetrazepin- und -tetrazozinderivaten. Arch. Pharm..

[41] Gilchrist T.L., Wasson R.C. (1987). Intramolecular cycloaddition of azoalkenes derived from terminal alkenoic and alkynoic acids. J. Chem. Soc. Perkin Trans. 1.

[42] Gresham T.L., Jansen J.E., Shaver S.W., Bankert R.A., Fiedorek F.T. (1951). β-Propiolactone. XI. Reactions with ammonia and amines. J. Am. Chem. Soc..

[43] Ottaway J.H. (1962). A preparation of d(−)-β-hydroxybutyric acid. Biochem. J..

[44] Boekelheide V., Goldman M. (1954). A synthesis of 3-bromoperinaphthanol-7. J. Am. Chem. Soc..

